# Hydrogen (H_2_) Recovery From Hydrogen Sulfide (H_2_S): Current Technologies, Challenges, and Future Outlook

**DOI:** 10.1002/advs.202523530

**Published:** 2026-05-25

**Authors:** Divyesh Cirikonda, Astrid Campos‐Mata, Sehmus Ozden, Shreyasi Chattopadhyay, Pulickel M. Ajayan

**Affiliations:** ^1^ Department of Materials Science and NanoEngineering Rice University 6100 Main Street Houston Texas USA; ^2^ Aramco Americans, Aramco Research Centre‐Houston 16300 Park Row Houston Texas USA

**Keywords:** electrocatalysis, H_2_ production, H_2_S splitting, photocatalysis, plasma‐assisted decomposition, thermocatalysis, waste to energy

## Abstract

Hydrogen sulfide (H_2_S) can be transformed into hydrogen (H_2_) through several chemical and catalytic processes, offering a promising route for both waste treatment and clean H_2_ production. This colorless, flammable, and toxic gas is found abundantly in swamps, volcanoes, hot springs, sewages, other natural gas fields, and even in refineries and during some industrial processes. A very low concentration of H_2_S could cause adverse health impacts and even fatality. Therefore, removal of H_2_S is necessary. Moreover, decomposition of H_2_S produces H_2_ which is being considered as one of the most promising sustainable clean energy solutions. The by‐product, sulfur, holds additional value through its diverse industrial applications and contribute to the circular economy. Numerous techniques, including thermal, thermo‐catalytic, photocatalytic and electrocatalytic approaches, are employed for H_2_S decomposition. This review provides a comprehensive discussion on recent progress on H_2_S splitting via different methods, including thermocatalysis, photocatalysis, electrocatalysis and plasma‐assisted decomposition to guide innovations in this highly promising yet unexplored areas. Moreover, energy requirement, economic viability and current market status are also explored. Finally, key challenges and future perspectives of this highly promising research area are discussed, directing great potential for future explorations.

## Graphical abstract

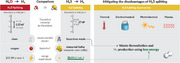

## 1 Introduction

Hydrogen (H_2_) is a clean, alternative energy resource that can lead the effort to transition the society from fossil‐fuel dependence to carbon‐neutral systems, thereby addressing the issues related to global warming and climate change. Among several strategies used in producing H_2_, photo and electrochemical water splitting are considered as the most viable approaches [1, 2, 3, 4, 5]. Additionally, to avoid thermodynamic and kinetic constraints associated with water splitting, continuous research efforts are being made in search of alternative H_2_ sources. In that scenario, H_2_S conversion to H_2_ is a promising alternative to conventional water splitting for H_2_ production. This approach also presents both environmental and thermodynamic advantages [6]. H_2_S is a colorless toxic gas known for its strong rotten egg odor. It is naturally released in the air from sewage, swamps, manure gas, hot springs, geysers, and volcanoes. The toxic gas is also found in industries such as the oil and gas, food processing, paper mills, tanneries, geothermal power plants, and wastewater treatment plants [7]. Air concentrations of H_2_S from natural sources typically range from 0.00011 to 0.00033 ppm, while urban areas generally see levels below 0.001 ppm. H_2_S can remain in the atmosphere for about 1 to 42 days, depending on the season, and can transform into sulfur dioxide and sulfates. Partial pressures exceeding 0.05 H_2_S are considered corrosive [8]. H_2_S can be released into water through industrial liquid waste or natural events. Typically, wells with H_2_S concentrations of 10 ppm or higher can be classified as sour [9]. Surface water usually has very low H_2_S concentrations due to its tendency to volatilize, but it can be present in groundwater, where levels are typically below 1 ppm. H_2_S can also enter the soil through atmospheric deposition or spills. While H_2_S is involved in some biochemical and environmental processes, it is primarily noted for its toxicity and associated health risks. Even low levels of exposure can lead to respiratory irritation, and higher concentrations can be life‐threatening, potentially causing loss of consciousness or death [10]. H_2_S poses significant environmental and industrial challenges that require careful management, often complex and expensive. Due to its environmental impact and the need for strict regulations, removal of H_2_S is crucial for maintaining safety in both natural and industrial settings [11].

In this scenario, producing H_2_ from H_2_S is a smart and cost‐effective way to meet the growing demand for environmental remediation and clean energy. In addition, the potential of H_2_ to serve as a key player in the transition to a low‐carbon economy is increasingly being explored across various sectors [12]. This work is also aligned with the United Nations Sustainable Development Goals, particularly SDG 7 (Affordable and Clean Energy), SDG 9 (Industry, Innovation and Infrastructure), and SDG 13 (Climate Action), as it supports the development of cleaner H_2_ production pathways, promotes innovative sulfur valorization, and contributes to the mitigation of environmental impacts associated with H_2_S emissions [13]. Notably, the United Nations Sustainable Development Goals (UN SDG), UN SDG 7 (clean and affordable energy), and UN SDG 12 (responsible consumption and production) collectively emphasize improved energy and resource efficiency, effective waste management, and the promotion of a circular economy, thereby contributing to climate change mitigation and environmental sustainability. H_2_ production from water electrolysis requires 9 tons of fresh water for 1 ton of H_2_. Pursuing this would contradict UN SDG 6, which targets clean water availability. In that context, H_2_ production from H_2_S allows for exploring waste‐to‐H_2_ production without affecting interconnected development goals [14, 15]. Therefore, it not only addresses the environmental issues but also participates in the research area of alternative energy resources. From transportation to industrial processes, H_2_ offers innovative pathways that could reshape our energy landscape for a more sustainable future. Since the only product produced after H_2_ burning is water, hydrogen‐powered vehicles can lower greenhouse gas emissions and improve air quality in cities [16]. In industrial settings, H_2_ is widely used to produce ammonia, a key component of fertilizers supporting global food production [17]. Additionally, in metallurgy, H_2_ is used to produce metals with a smaller carbon footprint. These advancements help industries meet environmental goals and adapt to changing regulations, while also positioning H_2_ as an effective energy alternative, addressing the challenges of renewable energy.

**FIGURE 1:**
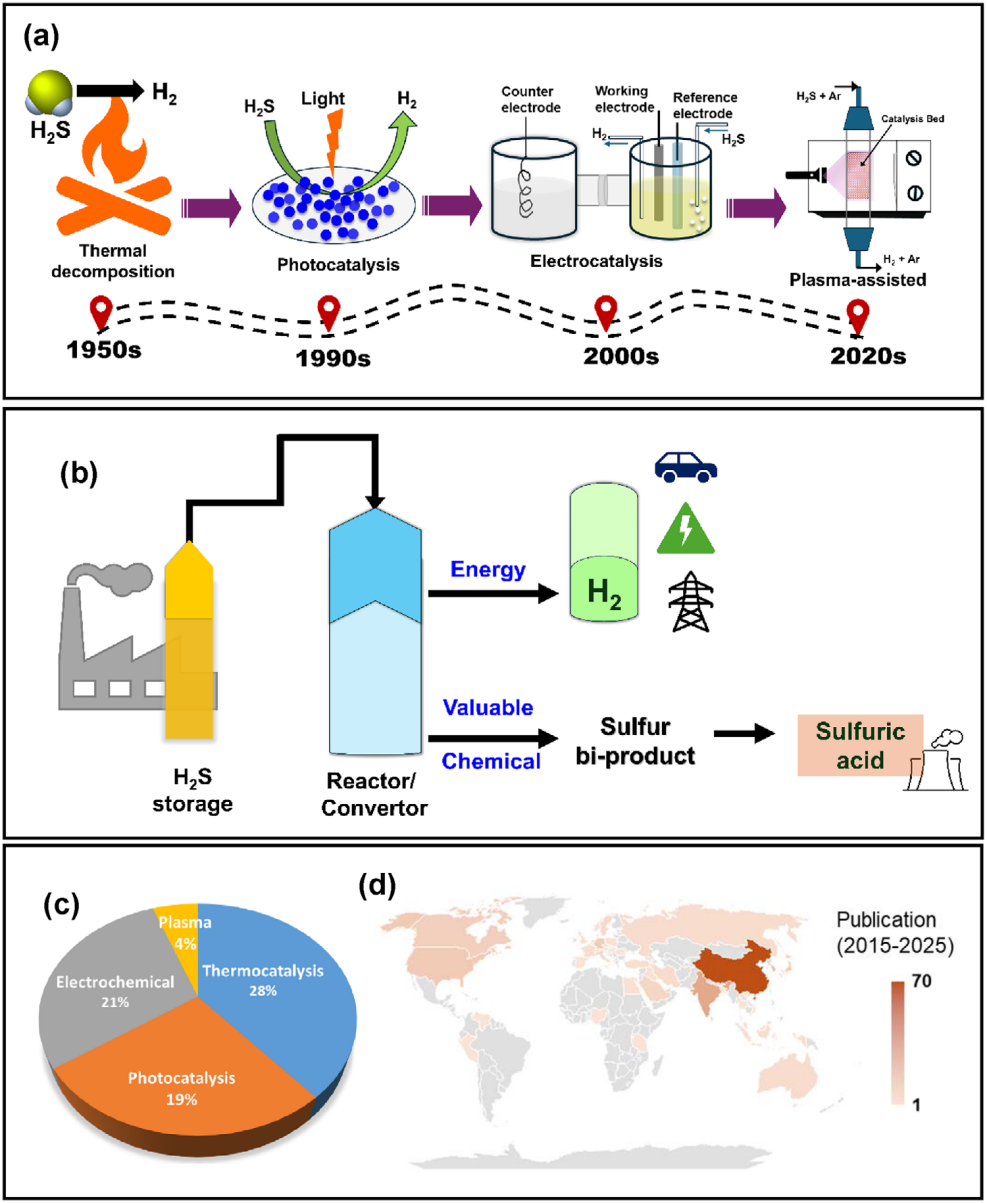
(a) Conversion approaches for H_2_ generation from H_2_S, (b) Scheme showing route to valorization of H_2_S, (c) Pie chart showing H_2_S splitting to H_2_ using various techniques, and (d) Country‐wise publication trend on H_2_S splitting. (c) and (d) are generated from the cumulative publication on H_2_S splitting over the years 2015 to 2025.

Over the years, several research studies have been done to explore the H_2_S conversion possibilities and attain higher efficiency [18, 19, 20]. Starting with the thermal decomposition, current methods of H_2_S conversion include photocatalysis, electrocatalysis, and plasma‐assisted decomposition (Figure 1a). The prospect of an achievable circular economy from H_2_S splitting is shown schematically in Figure 1b. Furthermore, a survey on publications has been conducted on Scopus search to understand the global research interest in this topic. Figure 1c,d is generated by considering the number of publications over the past 10 years on the research area of H_2_S splitting for H_2_. Figure 1c represents the rate of publication under the methods that are being explored for H_2_S conversion. Figure 1d illustrates the number of publications in different countries for the past 10 years, which shows a significant global urge for H_2_S splitting. However, there is still no industrial‐level application of the utilization of H_2_S for H_2_ production. The challenges lie in the corrosive nature and low H_2_ storage capacity of H_2_S. Therefore, the overall analysis demonstrates the wide interest of the scientific community in this field and conveys the relevance of the review. Moreover, our review addresses an important gap in the current literature. Most recent reviews are limited to a single H_2_S splitting pathway, such as chemical, thermal, photochemical, electrochemical, or plasma‐assisted processes, without providing a comprehensive cross‐comparison of these approaches [21, 22, 23]. Additionally, detailed discussions on reaction mechanisms, existing bottlenecks, and future research directions to address these challenges are often lacking. By presenting an integrated critical analysis of all major H_2_S‐splitting strategies, our review captures the broader momentum in this area and clearly establishes the significance and timeliness of the topic. This review aims to summarize the important aspects of the state‐of‐the‐art H_2_S splitting research, the significance of H_2_S to H_2_ conversion, including the energy and cost demands, recent research progress, and market studies. The evolution of different conversion approaches, including all the possible routes such as thermal/thermo‐catalytic, photochemical, electrochemical, and plasma‐assisted decompositions are explored with their recent research progress. The thermodynamic aspect of the H_2_S decomposition is discussed along with catalyst design. A detailed discussion on electrocatalytic splitting of H_2_S to H_2_ is presented, incorporating the mechanistic aspect. Finally, market analysis, current challenges, and future directions are discussed and highlighted, offering insights to the researchers and guiding them toward continued progress in the field.

## 2 Significance of H_2_ Production From H_2_S

Producing H_2_ from H_2_S offers an efficient and economically viable approach to address the rising demand for clean energy. Converting H_2_S to H_2_ provides a pathway to recycle waste H_2_S while recovering valuable sulfur and creating clean energy sources. Unlike the traditional method of steam reforming natural gas, which can be expensive and energy‐intensive, H_2_S offers several advantages. The price of natural gas can be volatile, influenced by market dynamics and geopolitical factors. In contrast, H_2_S is often available as a byproduct of various industrial processes, such as oil refining and natural gas processing. In addition, removal and treatment for H_2_S have become a concern owing to its toxicity. Producing H_2_ on‐site at petrochemical and refinery plants can lead to additional economic benefits. On‐site production minimizes transportation costs, which can be significant when moving H_2_ from centralized production facilities to end users. By generating H_2_ where it is needed, companies can streamline their operations, reduce logistical challenges, and improve supply chain efficiency. This localized production can also enhance energy security, allowing facilities to be less reliant on external H_2_ suppliers and their associated risks. Transforming H_2_S into H_2_ and sulfur can effectively tackle waste management issues, thus reducing environmental risks. This allows the development of an effective industrial waste and energy management system, effectively minimizing waste production, and a way to secure additional revenue. Innovations in H_2_ technologies, such as fuel cells and H_2_‐powered vehicles, create new opportunities for businesses that can supply this essential resource efficiently and sustainably. Companies can potentially generate revenue from the sale of recovered sulfur, which is in demand for agricultural fertilizers, chemicals, and other applications. This dual benefit of waste reduction and revenue generation enhances the overall economic viability of the process. Additionally, converting H_2_S can help fight global warming by lowering greenhouse gas emissions, making it a greener option. Therefore, using H_2_S to produce H_2_ saves money, supports industries, and helps protect the environment, paving the way for a more sustainable energy future [24].

## 3 Energy Requirement for H_2_S Splitting for H_2_ and Circular Economy

**FIGURE 2:**
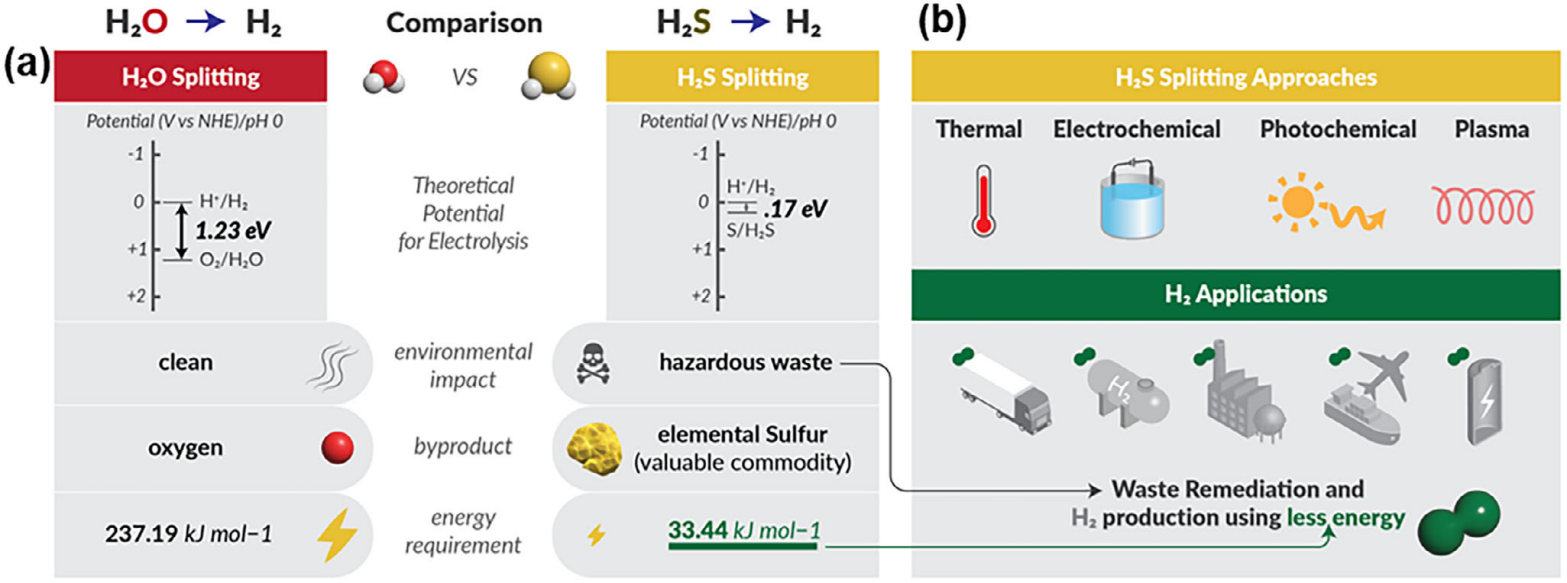
Summary of viability of H_2_ production via H_2_S splitting over H_2_O splitting: (a) shows the low energy requirement and valorization for H_2_S, (b) potential methods of H_2_S Splitting for H_2_ and sustainable utilization of H_2_.

The production of H_2_ from H_2_S is a more energy‐efficient process compared to other common methods, such as water splitting. For instance, most H_2_ is currently produced via steam methane reforming (SMR), a highly endothermic reaction (∆H_298K_ = 206 kJ mol^−1^) [25]. In contrast, H_2_S splitting, while also endothermic, requires significantly less energy (∆H_298K_ = 20 kJ mol^−1^) [26]. This makes H_2_S splitting, whether achieved through thermal, photocatalytic, electrocatalytic, or plasma‐assisted methods, a thermodynamically more favorable process than SMR. Green H_2_ can also be obtained by water splitting using photocatalytic and electrocatalytic methods. However, H_2_S splitting is more energy‐efficient because the H‐S bond dissociation energy is lower than that of H_2_O (∆H_298K_ = 285 kJ mol^−1^) [27]. The mechanistic differences between H_2_O and H_2_S splitting come from the fundamental chemical properties of the two compounds, and dictate their distinct energy requirements [28], challenges, and areas of usefulness. The core distinction lies in the bond strengths and polarity. Water, with its highly electronegative oxygen atom, forms strong polar O‐H bonds and exhibits extensive intermolecular H_2_ bonding. This inherent stability necessitates a substantial energy input to break these bonds and evolve oxygen, making the oxygen evolution reaction (OER) the primary energy barrier in water splitting. In contrast, H_2_S features a larger, less electronegative sulfur atom, resulting in weaker and less polar S‐H bonds and negligible H_2_ bonding. This chemical reality translates to a significantly lower thermodynamic energy requirement for H_2_S splitting; a theoretical electrolytic voltage of approximately 0.14–0.17 V compared to 1.23 V for water, making it an energetically attractive pathway for H_2_ generation. Thermodynamically, H_2_O dissociation is energy‐intensive due to the stability of the H‐O bond (ΔG° ≈ +237.19 kJ mol^−1^ at 298 K). The stoichiometric reaction: H_2_S → H_2_ + 1/2 S_2_, is thermodynamically (ΔG° ≈ +33.44 kJ mol^−1^ at 298 K), and requires less external energy input to overcome kinetic and thermodynamic barriers [29, 30, 31]. Figure 2a summarizes the viability of H_2_ production via H_2_S splitting over H_2_O splitting. Moreover, being a potential H_2_ source, H_2_S has the lowest enthalpy of formation (−20 kJ mol^−1^) in comparison to the other sources like CH_4_ (−74.9 kJ mol^−1^), H_2_O (−285.4 kJ mol^−1^), NH_3_ (−45.9 kJ mol^−1^), and metal hydrides (∼ −100 to −190 kJ mol^−1^) [31, 32].

## 4 Methods of H_2_ Production From H_2_S

H_2_S can be converted to H_2_ via several chemical and catalytic routes. Figure 2b shows the potential ways to dissociate H_2_S and the utility of the produced H_2_. Table 1 shows the comparative table summarizing H_2_S to H_2_ production via thermal, photo, electrochemical, and plasma‐assisted approaches [33, 34, 35, 36]. The Claus process is the dominant industrial process to decompose H_2_S. In the 19th century, industries processing natural gas and petroleum noticed that H_2_S was a major contaminant, toxic, corrosive, and environmentally hazardous. Therefore, with an urge to convert H_2_S into something safer and more useful product, Carl Friedrich Claus, a German chemist, invented the Claus process to convert H_2_S into elemental sulfur and water in 1883. The original Claus process was a single‐step process toward thermal decomposition of H_2_S in S and H_2_O. For a better conversion rate, the oil and gas industries started modifying Claus's original process. However, while it achieves high sulfur recovery efficiency (95%–98%), its H_2_ recovery is minimal or null. Given the growing interest in H_2_ as a clean energy carrier, there is increasing economic motivation to develop processes that efficiently recover both H_2_ and sulfur as valuable commercial products. Thermal decomposition has been explored at the pilot scale in industrial settings. The H_2_ recovery efficiency of this method ranges from 40–70%, while sulfur recovery efficiency varies between 80% and 100%, depending on heat recovery strategies and catalyst use. However, this process faces several challenges, including high energy demands due to the need to reach temperatures around 1000°C [37]. In contrast, photocatalytic H_2_S splitting is a sustainable approach converting it into H_2_ and elemental sulfur under mild conditions, utilizing solar energy, a clean, abundant, and sustainable energy source. This approach not only mitigates the environmental threat posed by H_2_S but also contributes to green H_2_ production, aligning with the goals of the circular economy and carbon‐neutral energy systems [38, 39, 40]. Electrolysis has also attracted interest due to its lower operating temperatures and high recovery efficiencies for both H_2_ and sulfur (80%–100%). Recent research has reported recovery efficiencies exceeding 95% [41]. Another report on solar‐driven electrochemical H_2_S splitting showed 12% solar to H_2_ conversion efficiency in a diaphragm electrolyte reactor [42]. Despite these promising results, electrocatalytic splitting faces significant challenges, including sulfur deposition, electrode passivation, and the absence of large‐scale demonstrations [43]. The lack of sufficient advancement in research and development has hindered this method's scalability. Finally, plasma‐assisted decomposition has emerged as a high‐efficiency (70%–90%) prospect with lower energy requirements than thermal methods, though it has yet to be tested in industrial applications [44]. In this section, all these processes are discussed further in detail.

**TABLE 1:** Approaches for H_2_ production from H_2_S.

Approach	Operating condition	H_2_ yield with catalyst	Environmental impact	Key advantage	Challenges	Current research status
Thermal	High Temperature (700°C–1200°C)	∼95%	Moderate CO_2_, some SO_x_, sulfur byproduct	Industry proven; simple scale‐up	Large energy input; emission concerns	Industrial‐scale for sulfur recovery (Claus process, direct decomposition)
Photocatalysis	UV/Visible light; ambient.	∼67%	Very low with renewables; elemental S recovery	Clean, renewable, valorizes H_2_S waste	Catalyst stability/efficiency under actual sunlight remains under study. S deactivates the catalyst	Early‐stage, mainly lab/pilot studies;
Electrocatalysis	Ambient/low Temperature, flow cell,	∼99%	Very low with renewables; S by product	Lower energy, ambient, scalable	technical challenge: S passivation Electrode fouling by S; cell design	Advanced lab phase with promising future application in large scale
Plasma‐assisted	Non‐thermal/MW/arc plasma, ambient–1000°C	60%–98% (lab/pilot, best >90%)	Very low with renewables, elemental S byproduct	High conversion, good for dilute H_2_S	High energy; device/catalyst durability	Active research, promising results for dilute/industrial H_2_S

### 4.1 Thermo‐Catalytic Conversion of H_2_S for H_2_

The thermocatalytic decomposition of H_2_S into H_2_ and elemental sulfur is one of the promising approaches for sustainable H_2_ production while minimizing environmental risks associated with H_2_S emissions. Thermocatalytic conversion of H_2_S is a direct catalytic process that splits H_2_S into H_2_ and elemental sulfur (S_2_ or S_8_) at elevated temperatures (Equation 1) [45, 46, 47].

(1) $$2H_{2}S\to 2H_{2}+S_{2}$$

**FIGURE 3:**
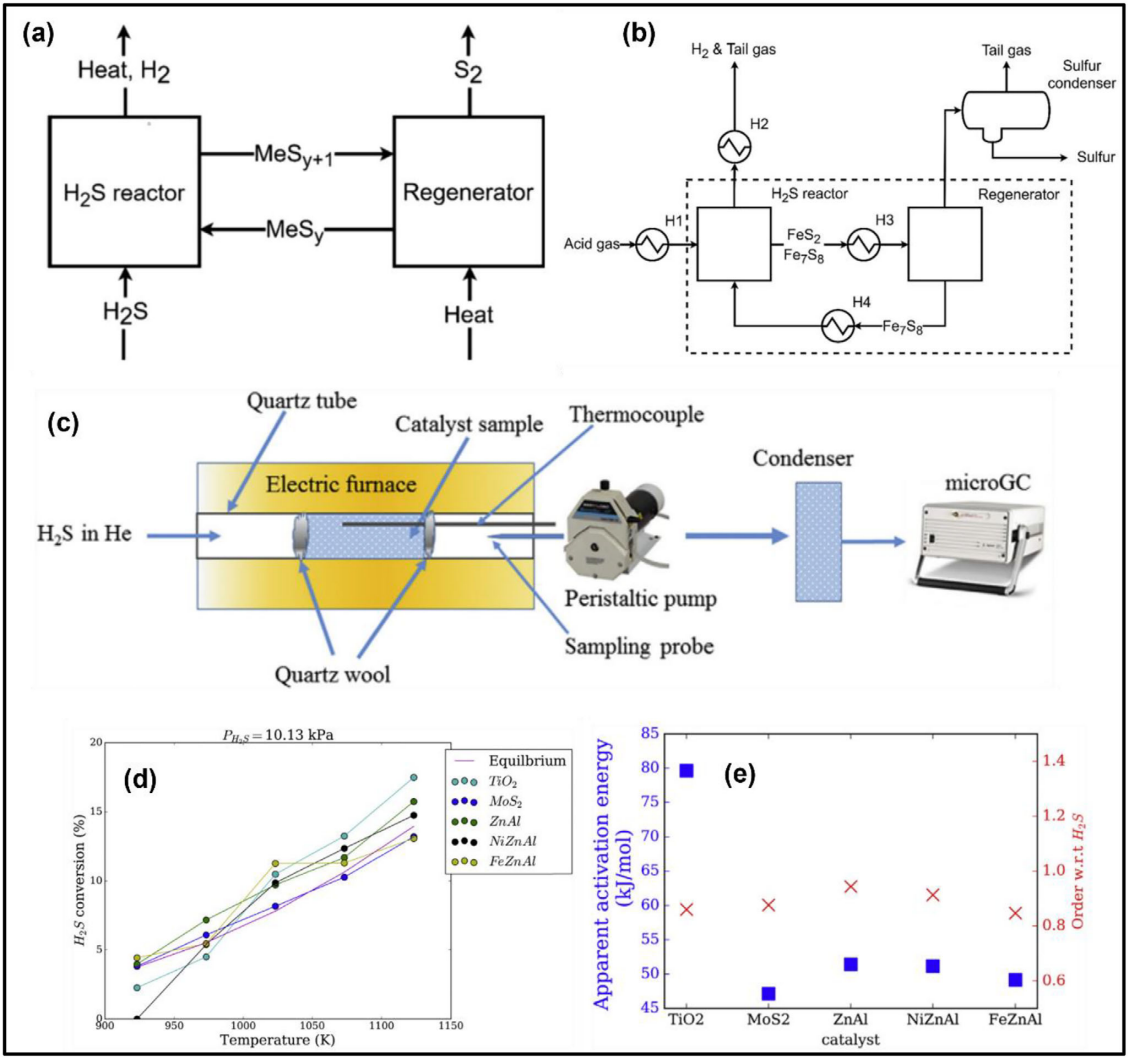
(a) Basic schematic of the SHR system using arbitrary metal sulfide (MeS) for sulfur and H_2_ production. (b) Schematic of the chemical looping configuration of the SHR system for sulfur and H_2_ recovery [54]. Copyright 2019, Elsevier (c) A schematic of the packed bed reactor facility. (d) H_2_S conversion at different temperatures using different catalysts and their comparison with the thermodynamic equilibrium yields. (e) Apparent activation energy (blue squares) and the order of H_2_S for H_2_ production (red crosses) for the different catalysts [46]. Copyright 2018, Elsevier.

This reaction is highly endothermic (ΔH° = +90.4 kJ mol^−1^) require high energy input to achieve significant conversion rates. The process has gained great attention as an approach for the conversion of H_2_S into valuable H_2_ while recovering elemental sulfur [48]. Thermal conversion of H_2_S without a catalyst, in general, happens at temperatures between 700°C and 1400°C. At such high temperatures, the reaction progresses in the gas phase, but it is limited by thermodynamic equilibrium [49, 50]. For example, under typical conditions, single‐pass H_2_S conversions are often limited to 22–26%, while advanced reactor designs and process intensification strategies can improve these numbers [32, 48]. Thermocatalytic conversion advances via homolytic cleavage of H_2_S, creating H_2_ and sulfur radicals. In the presence of catalysts (e.g., metal sulfides or oxides), the reaction can occur at lower temperatures with various mechanistic pathways, usually involving intermediate species such as disulfane (H_2_S_2_) or adsorbed sulfur species [51, 52]. Figure 3a illustrates a general schematic of sulfur‐hydrogen recovery (SHR), utilizing metal sulfides (MeS) as intermediates to produce both H_2_ and sulfur. Figure 3b shows a schematic of the chemical looping configuration of the SHR system for sulfur and H_2_ recovery [52, 53, 54, 55]. On the surface of catalysts, the process may be further facilitated by surface reactions, which lower the activation barrier for H_2_ release and sulfur recombination, as shown in the following Equations (2), (3), (4), (5), (6), (7), (8), (9), (10), (11) [33, 46, 52, 56].

H_2_S splitting on the catalyst surface:

(2) $$H_{2}S\to HS_{*}+H_{*}$$

H_2_ desorption:

(3) $$2H\to H_{2}$$

Recombination to S_2_:

(4) $$2S\to S_{2}$$

Overall:

(5) $$2H_{2}S\to 2H_{2}+S_{2}$$

On catalyst surfaces, the mechanism usually involves adsorbed species and intermediates:

(6) $$H_{2}S\left(g\right)\to H_{2}S^{*}$$

(7) $$H_{2}S^{*}\to HS^{*}+H^{*}$$

(8) $$HS^{*}\to H^{*}+S^{*}$$

(9) $$2H^{*}\to H_{2}\left(g\right)+*\;$$

(10) $$2S^{*}\to S_{2}\left(g\right)+*$$

(11) $$2HS^{*}\to H_{2}{S_{2}}^{*}\to H_{2}\left(g\right)+{S_{2}}^{*}$$

In thermo‐catalytic pathways that include metal, metal sulfides or oxides, the decomposition of H_2_S typically proceeds through a series of surface‐mediated steps, which lower the activation barrier and facilitate H_2_ and sulfur release [33, 54]. Initially, H_2_S molecules adsorb onto the electrophilic sites of the catalyst surface, such as those present on low‐sulfur metal sulfide clusters (e.g., MoS_2_ or WS_2_). Upon adsorption, a sequential hydrogen‐atom transfer process occurs, in which the hydrogen atoms from H_2_S are transferred to the surface of the catalyst, resulting in the formation of surface‐bound SH groups and ultimately leading to the release of molecular H_2_ [52]. Concurrently, the catalyst surface incorporates additional sulfur, transitioning from a low‐sulfur to a high‐sulfur state (e.g., from MS_3_ to MS_4_), with the high‐sulfur cluster containing an adsorbed singlet S_2_ moiety. The formation of surface intermediates such as disulfane (H_2_S_2_) has also been identified as a key step, where two adjacent adsorbed H_2_S molecules form an S‐S bond, releasing H_2_ and generating adsorbed S_2_. At elevated temperatures, the adsorbed S_2_ can desorb as elemental sulfur, thus regenerating the active catalyst sites. The overall process is energetically favorable, with relatively low reaction barriers under suitable catalytic conditions. This mechanistic understanding features the critical role of catalyst structure and surface chemistry for enhancing H_2_S conversion efficiency and selectivity. The efficiency and viability of thermocatalytic H_2_S decomposition are strongly influenced by the choice of the catalyst. Recent advancements have focused on improving conversion rates, lowering operating temperatures, and enhancing sulfur tolerance.

Transition metal sulfides are the most commonly studied catalysts for H_2_S decomposition, owing to their inherent sulfur tolerance and activity. Molybdenum disulfide (MoS_2_) and tungsten disulfide (WS_2_) in particular have been shown to be effective, especially at higher temperatures (≥600°C) [53]. Early studies by Chivers and others identified MoS_2_ as a top performer, maintaining catalytic activity in H_2_S atmospheres where many metals would become poisoned. For example, MoS_2_ and WS_2_ can achieve measurable H_2_ production at 550°C–600°C, whereas uncatalyzed H_2_S at those temperatures yields virtually no H_2_. The activity of MoS_2_ is attributed to its sulfur‐vacancy sites and edges that can bind and split H_2_S; a proposed mechanism involves H_2_S adsorption on Mo sites, formation of Mo‐S‐H intermediates, and H_2_ release via recombination of surface hydrogens. WS_2_ behaves similarly and, in some studies, showed higher activity than MoS_2_ below ∼600°C [57]. Metal sulfides of Ni, Fe, Co can also decompose H_2_S, but as noted, these often function via sulfidation–desulfidation cycles. For instance, nickel sulfide (Ni_3_S_2_/NiS) can produce H_2_ at 500°C–600°C by converting to higher sulfides and back. This “sulfur shuttle” mechanism means the catalyst progressively sulfides during H_2_S exposure and must be regenerated by releasing sulfur by heating in an inert gas. While such cycles can be leveraged in two‐step processes (discussed later), they are less ideal for steady single‐pass operation [57].

While metal sulfides dominate this field, certain metal oxides and composite materials have shown promise, especially at lower H_2_S concentrations or in resisting sintering at high temperatures. Metal oxides initially are not active for H_2_S splitting until they become sulfided in situ. For example, Kraia et al. introduced a series of ceria‐supported transition metal precatalysts, denoted as M/CeO_2_ (where M = Co, Ni, Fe, and Cu), using a feed gas composed of 1% v/v H_2_S in Ar (0.01 atm H_2_S). Among these, the cobalt‐based precatalyst exhibited the highest reactivity, achieving H_2_S conversions near thermodynamic equilibrium at temperatures ranging from 823 to 1123 K [58]. This enhanced performance was attributed to the formation of sulfided active phases during the activation stage. Variants with increased cobalt loading were also tested under water‐rich conditions (1% v/v H_2_S, 90% v/v H_2_, with Ar as the diluent), simulating the H_2_S/H_2_O inflow characteristic of Black Sea deep waters. The presence of steam improved both H_2_S conversion and H_2_ yield, although H_2_ generation via water splitting remained negligible. In addition to direct decomposition, H_2_S steam reforming occurred concurrently, further boosting H_2_ production while generating SO_2_ as a byproduct [58]. To better understand the effect of the catalyst on H_2_S splitting, Burra et al. [46] utilized a packed bed reactor (Figure 3c) for catalyst testing. In this reactor, gases (including the H_2_S feed) flow into the system where they pass through a bed of catalyst particles packed inside a heated quartz tube. The entire apparatus is designed for safely and precisely studying how well the catalyst works to break down H_2_S under controlled high temperatures. Using this packed bed reactor, they tested several catalysts (metals, oxides, and sulfides). It is important to note, as shown in Figure 3d, that all these catalysts exceed the predicted thermodynamic equilibrium limit. This enhanced performance is attributed to the in situ removal of sulfur (which solidifies outside the hot zone), shifting the reaction according to Le Chatelier's principle. In their study, they reported TiO_2_ with the highest conversion at temperatures above 1073 K. Figure 3e shows the apparent activation energy (blue squares) and the reaction order with respect to H_2_S (red crosses) for the same catalysts. Surface area and diffusion effects were not considered in this apparent activation energy calculation, as evidenced by the units of the H_2_ yield rate (per unit gram of catalyst). There is a clear distinction between the activation energy of TiO_2_ and that of the other catalysts.

Although catalytic thermal conversion of H_2_S for H_2_ production is a promising approach, several key challenges hinder its commercial feasibility. One of the main challenges is the development of stable catalytic processes, which faces hurdles at both the fundamental catalyst level and the process level. Catalyst deactivation due to sulfur poisoning is a primary issue. Most existing catalysts can initially decompose H_2_S but lose activity rapidly as sulfur accumulates on their active sites, blocking further reaction and necessitating frequent regeneration or replacement. This stability and durability problem is compounded by the need for catalysts that are not only active and selective [53].

Thermodynamic limitations are one of the other major challenges. H_2_S decomposition is an endothermic reaction, with equilibrium conversion limited at lower temperatures, requiring high energy input to achieve practical yields. Even with advanced catalysts, overcoming these thermodynamic barriers without excessive energy consumption remains a significant challenge. To overcome the thermodynamic constraints of H_2_S conversion, several strategies can be employed to shift the reaction equilibrium toward higher conversions. Continuous removal of H_2_ using hydrogen‐permeable membranes effectively drives the reaction forward by lowering product concentration.

Process scalability and integration can advance complicated industrial adoption. Large‐scale implementation requires robust reactor designs that can handle high H_2_S concentrations, efficiently separate and recover H_2_ and sulfur, and manage heat integration and recycling of unreacted gases. Additionally, the cost and durability of novel catalysts can support the economic feasibility for widespread use.

### 4.2 Photocatalytic and Photoelectrocatalytic Conversion of H_2_S for H_2_

Photocatalytic splitting of H_2_S utilizes solar or artificial light to drive the redox reaction at ambient temperature and pressure, thus potentially reducing the high energy cost of thermal or electrochemical routes. Thermodynamically, H_2_S decomposition is well fitted within the scope of photocatalysis. In the gas phase at 298 K, the standard free energy of reaction ΔG°_r_ = +73.2 kJ mol^−1^, which corresponds to ∼0.76 eV per H_2_S (≈0.38 eV per electron for a two‐electron process). Therefore, semiconductors with band gaps in the visible or even near‐IR range could be good photocatalysts, enabling better utilization of the solar spectrum. The idea of using light to decompose H_2_S emerged soon after the discovery of semiconductor‐based photocatalysis for water splitting.

**FIGURE 4:**
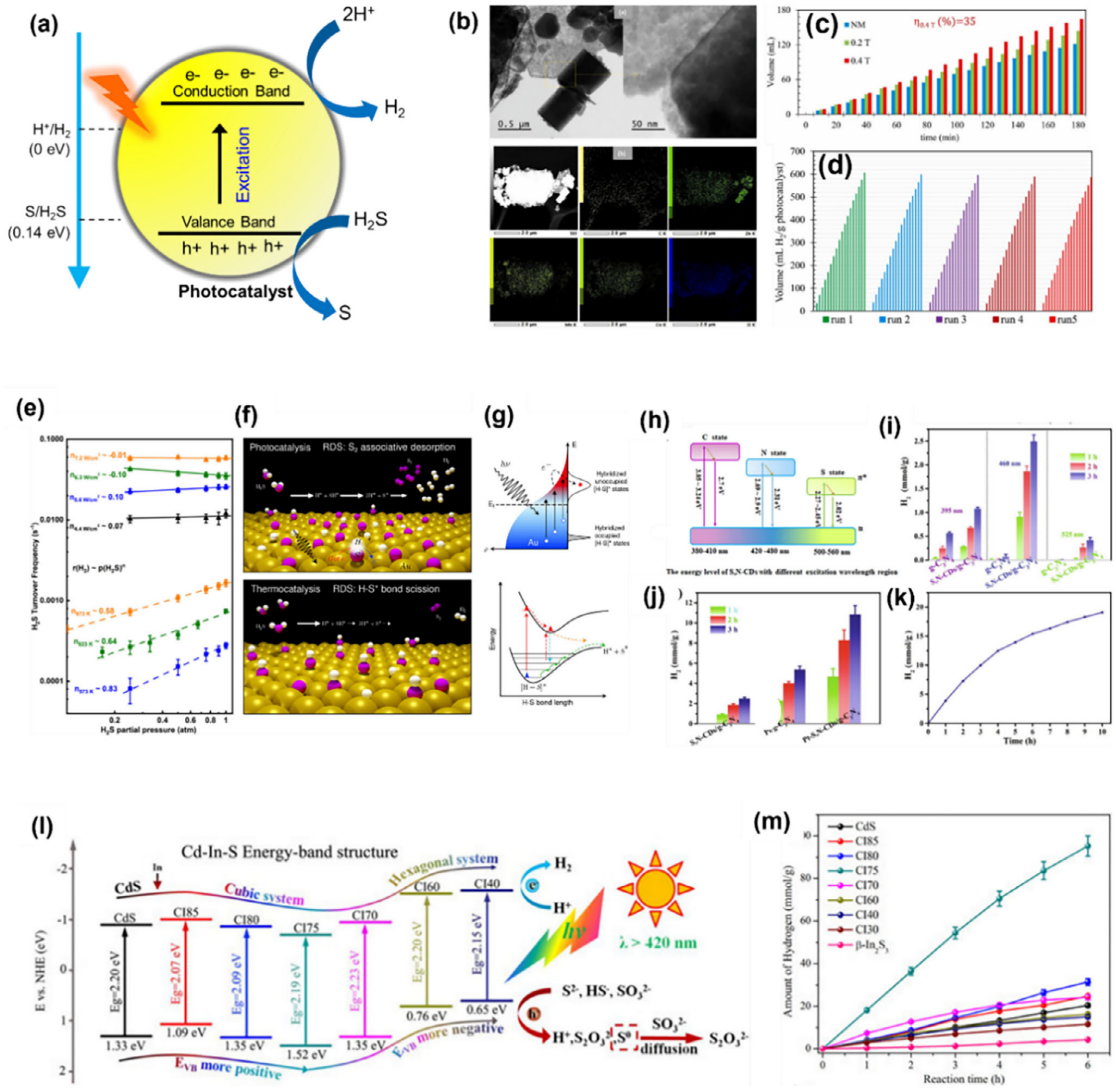
Photocatalytic conversion of H_2_S to H_2_: (a) Schematic showing the mechanism for photocatalysis; (b–d) rGO/CoMn_2_O_4_‐ZnO photocatalyst, the effect of applied magnetic field on the activity and cyclic stability, respectively [60]; Copyright 2025, Elsevier (e) Turnover frequencies for Au surface as a function of H_2_S partial pressure under different incident light power and dark thermocatalysis [61]; (f) photo‐ and thermocatalytic H_2_S decomposition on the Au surface [61]; (g) role of plasmon‐induced hot electrons of Au for activating the H‐S bond in HS* surface intermediates [61]; Copyright 2025, American Chemical Society. (h) The energy level diagram of S,N‐CDs with different excitation wavelength regions [62]; (i) g‐C_3_N_4_ and S,N‐CDs/g‐C_3_N_4_ under irradiation by three LED lamps (λ = 395 nm, 160 mW/cm^2^; λ = 460 and 525 nm, 150 mW/cm^2^ [62]; (j) S,N‐CDs/g‐C_3_N_4_, Pt/g‐C_3_N_4_ and Pt‐S,N‐CDs/g‐C_3_N_4_ (1wt%) [62]; (k) Stability test of Pt‐S,N‐CDs/g‐C_3_N_4_ (λ = 460 nm, 150 mW/cm^2^) [62]; Copyright 2021, Elsevier (l) Energy band structure of Cd_x_In_1‐x_S (x = 100, 85, 80, 75, 70, 60, 40, 30, and 0) solid solutions and photocatalytic process of splitting H_2_S [63]; (m) Photocatalytic H_2_ production rate by Cd_x_In_1‐x_S (λ > 420 nm) [63]. Copyright 2018, Wiley.

The overall photocatalytic reaction in the presence of the photocatalyst and light irradiation is represented in Figure 4a, and the following reaction steps are involved as follows. Upon illumination with photons of energy equal to or greater than the bandgap of the photocatalyst, electron‐hole pairs are generated. The photogenerated electrons participate in the reduction of protons to produce molecular H_2_, while the holes oxidize H_2_S, forming sulfur (S^0^) or sulfite/sulfate species depending on reaction conditions. The simplified redox half‐reactions are the following (Equations (12), (13), (14)) [36].

Oxidation (at valence band):

(12) $$H_{2}S\to S+2H^{+}+2e^{-}$$

Reduction (at conduction band):

(13) $$2H^{+}+2e^{-}\to H_{2}$$

Overall reaction:

(14) $$H_{2}S\to H_{2}+S$$

Several critical parameters govern the efficiency of H_2_S photocatalytic splitting. Efficient separation of electron‐hole pairs and rapid charge transport are essential to minimize recombination losses. Direct photolysis of H_2_S can be achieved under UV‐C irradiation with a wavelength of 254 nm [59]. For photocatalysis, the catalyst material should possess suitable band edge positions. Under the liquid phase reaction condition, the difference between the redox potentials of H^+^/H_2_ is 0 V, and S/H_2_S is only 0.14 V.

Therefore, the conduction band minimum is more negative than 0 V vs. NHE for H^+^ reduction $\left(E_{\mathrm{CB}}<E_{H^{+}/H_{2}}\right)$ and the valence band maximum is more positive than the oxidation potential of H_2_S $\left(E_{\mathrm{VB}}<E_{H_{2}S/S^{0}}\right)$. Surface area and chemical stability under corrosive conditions also affect the catalytic activity and efficiency.

Early studies in the 1970s explored UV‐activated semiconductor photocatalysts such as TiO_2_ for H_2_S decomposition; however, the wide bandgap of TiO_2_ (∼3.2 eV) limited its applicability under solar radiation. The breakthrough came with the introduction of narrow‐bandgap metal sulfide photocatalysts. During 1977–1980, early experiments with CdS, ZnS, and MoS_2_ powders showed visible‐light‐driven decomposition of H_2_S into H_2_ and S^0^. These works demonstrated that sulfide semiconductors could absorb visible light (∼2.0–2.5 eV bandgaps) and directly oxidize S^2−^ to elemental sulfur while reducing protons to H_2_. One of the initial works on photo‐splitting of H_2_S reported 40% conversion in H_2_S/CO_2_ system for splitting H_2_S to sulfur and H_2_ [64]. Sulfide‐based photocatalysts are attractive for H_2_S photo‐reforming as they exhibit better tolerance to sulfur‐rich environments and can sometimes undergo self‐regeneration. Semiconductors like TiO_2_, CdS, ZnS, and MoS_2,_ in particular, have been extensively studied due to their suitable band structure for visible light absorption and favorable conduction band position for proton reduction. Direct splitting of H_2_S to H_2_ and S under visible light has been achieved on CdS catalyst, which also showed good stability [65]. However, photocorrosion of the catalyst in aqueous media is a significant drawback. To enhance charge separation and stability, heterojunctions (e.g., CdS/TiO_2_, g‐C_3_N_4_/ZnIn_2_S_4_), co‐catalyst decoration (e.g., Pt, Ni), and surface passivation strategies have been developed. Additionally, the use of sacrificial electron donors such as Na_2_S, Na_2_SO_3,_ which acted both as hole scavengers and sulfur stabilizers are considered to mitigate the photocorrosion issue. CdS has been the most extensively studied photocatalyst for H_2_S splitting due to its suitable bandgap (2.4 eV) and conduction band edge potential (−0.5 V vs. NHE). To address its photo‐corrosion in aqueous environments, especially under oxidative conditions and subsequent loss of activity and formation of Cd^2^^+^ ions, researchers explored co‐catalyst loading (e.g., Pt, Ni, RuO_2_) [66]. ZnS has a larger bandgap (∼3.6 eV) but offers better chemical stability. Doping with transition metals (e.g., Cu, Mn) can narrow the bandgap and extend light absorption into the visible range. Ternary Metal Sulfides (e.g. AgIn_5_S_8_, ZnIn_2_S_4_, CuInS_2_) materials have attracted attention for their tunable optical properties, layered structures, and better photocorrosion resistance [67, 68]. ZnIn_2_S_4_, for instance, has demonstrated enhanced H_2_ evolution rates from H_2_S in the presence of hole scavengers [69]. Hollow microsphere ZnIn_2_S_4_/CdS composites with Pt nanoparticles achieved >90% H_2_ selectivity under visible light. The superior photocatalytic activity was achieved for a novel rGO/CoMn_2_O_4_(p)‐ZnO(n) nanocomposite, where the enhancement in activity was attributed to improved light absorption, anticipation of charge recombination, and higher reactant‐adsorption capacity. The activity of the photocatalyst was further boosted by 35% after applying an external magnetic field to the photoreactor (Figure 4b). This boosting effect was attributed to the improved magnetization of the p‐n nanocomposite in the presence of rGO and showed enhancement in sulfide sorption capacity and subsequent H_2_ production ability (Figure 4c,d) [60]. Layered transition metal dichalcogenides such as MoS_2_ and WS_2_ are valued for their high surface area, visible light absorption, and stability in sulfur‐rich environments. Their role as co‐catalysts and main catalysts in hybrid systems is also well‐documented. Among non‐sulfide materials, transition metal oxides with transition metal at d^0^ or d^10^ configuration are found to be promising. However, they have a wide band gap and are mostly active under UV light irradiation. To minimize the band gap, a combination with other cocatalysts such as noble metals with plasmonic characteristics or other solar light‐responsive materials are considered. Direct H_2_S decomposition to H_2_ and sulfur was achieved using SiO_2_‐supported Au nanostructured catalyst [61]. Comparison of microkinetics involved in thermal and photocatalytic decomposition processes (Figure 4e) revealed that light illumination significantly affected the kinetics and energetics. Directing photoexcitation of the metal states induced nonthermal dissociation and desorption that proposed to modify the coverage of surface species and thus the reaction pathway (Figure 4f,g). Heterojunction formation (e.g., CdS/TiO_2_, CdS/g‐C_3_N_4_) to suppress recombination and enhance stability. Defect‐engineered g‐C_3_N_4_ with MoS_2_ co‐catalysts enabled long‐term operation with minimal sulfur fouling. S, N co‐doped carbon dots (S,N‐CDs)/graphitic carbon nitride (g‐C_3_N_4_) nanosheet (Figure 4h–k) was found to show efficient photocatalyst for the decomposition of H_2_S [62]. One of the major challenges is the deactivation of photocatalysts due to the accumulation of sulfur species on active sites, which blocks light absorption and hinders surface reactions. The fundamental redox reactions involved in H_2_S photocatalytic splitting result in the formation of elemental sulfur and polysulfides. Studies using in situ spectroscopy and surface analysis techniques e.g., XPS, Raman, SEM have shown that catalyst deactivation is primarily due to sulfur fouling, which blocks active sites and reduces light penetration.

In case of Cd_x_In_1‐x_S system (Figure 4l,m), making the valence band more positive via band gap engineering has been proven to eliminate the sulfur deposition on catalyst surface, therefore resulting in long‐term stability [63]. To mitigate this issue, flowing gas‐phase reactors have been designed to continuously remove H_2_ and S, reducing fouling [59, 77].

Another approach is to use oxidative environments or electron acceptors (e.g., O_2_, Fe^3^^+^) that can convert S^0^ to soluble sulfate or sulfite, keeping the catalyst surface clean [74]. Formation of active species such as HS^−^ or S^2−^ depends on pH and significantly affects the reaction mechanism; therefore pH of the reaction medium also controls the oxidative behavior of H_2_S. Table 2 summarizes the activity profile of photocatalysts explored for H_2_S splitting for H_2_.

**TABLE 2:** Literature reports on Photocatalytic H_2_S splitting for H_2_.

Catalyst	Light source	H_2_S conversion efficiency	Key mechanism	References
CuS	20 W lamp, UV‐C	66% in 1h	Cu vacancies and plasmon‐enhanced charge separation	[59]
Mo‐modified TiO_2_ (gas‐phase)	Visible	85% H_2_ yield in 90 min (gas phase catalysis with H_2_S)	Mo↔MoS_2_ self‐regenerative cycle, minimizing sulfur poisoning	[70]
NiS_2_/CdS	Visible (λ > 420 nm)	44.39 mmol h^−1^ g^−1^	Cocatalyst NiS_2_ improves charge transfer and sulfur tolerance	[71]
Cu_2_S@CdS p‐n heterojunction	Visible solar simulator	32.5 mmol h^−1^g^−1^	Strong internal electrostatic field promotes electron migration	[72]
MoS_2_/TiO_2_ composite	Visible	201.84 µmol g^−1^ h^−1^	MoS_2_ increases stability and adsorption of H_2_S fragments	[73]
Anatase/TiO_2_(B) Nanotubes	UV (365 nm)	75 µmol h^−1^ g^−1^	Operates in low humidity; holes + O_2_^−^‐ radicals active	[74]
CdS@MoS_2_ core‐shell	Visible	49.8 mmol h^−1^ g^−1^	Type‐II heterojunction efficiently separates charges	[75]
In situ CdS/Cd(OH)_2_	Visible	12.52 mmol h^−1^ g^−1^	Cd(OH)_2_ improves surface hydro‐activity and sulfur binding	[76]
S,N‐CDs/g C_3_N_4_	Visible	10 mmol h^−1^ g^−1^	Carbon dots (CDs) enhance light absorption and electron mobility	[62]
CdS and ZnS powders	UV (365 nm)	0.9–3.0 mmol h^−1^ g^−1^	Direct semiconductor H_2_S splitting, CdS most active	[65]

A modern photoelectrocatalysis/photoelectrochemical (PEC) H_2_S splitting method is inherently interdisciplinary. These routes can be effective, but they often remain energy‐intensive or limited by sluggish anodic kinetics and sulfur management, and they don't leverage modern advances in semiconductor interfaces, photon management, and operando mechanistic tools. A clear emerging gap is the lack of PEC H_2_S splitting, which integrates solar‐driven charge generation with electrocatalytic surface reactions to reduce the required electrical bias and improve selectivity toward valuable sulfur products. PEC offers additional “knobs” (illumination intensity, wavelength tuning, junction fields, surface states) to tune reaction pathways. Not many reports are available on this area of research because it requires an integrated design. Recent work explicitly proposes photoelectrocatalytic strategies for simultaneous H_2_S remediation and H_2_ generation, showing this is an actively developing direction rather than a purely conceptual add‐on. In this work, Reis et al. have successfully shown degradation of 8.2 mg S with a Coulombic efficiency of 3600 mg S Ah^−1^ for H_2_S oxidation and a Faradaic efficiency of 60% for H_2_ evolution at an applied current density of 0.33 mA cm^−2^. Illumination with a 10 W high‐power blue LED significantly increased charge separation and reduced the cell potential, resulting in higher energy efficiency [78]. In another work, Zhong et al. demonstrated a solar‐to‐chemical conversion process using a photoelectrochemical cell without external bias for selective oxidation of H_2_S to produce hydrogen peroxide (H_2_O_2_) and sulfur [79]. Hematite has been reported for H_2_ production from water spitting. Lora et al. investigated spray‐pyrolyzed Sn(IV)‐doped α‐Fe_2_O_3_ photoanodes for photo‐assisted splitting of H_2_S in alkaline aqueous solutions, producing polysulfide (S_n_^2−^) ions together with H_2_ at the cathode. At an applied electrode potential of 1.07 V (RHE) and an irradiance of 5.6 kW m^−2^, stable photocurrents of 11 A m^−2^ (2 × 10^−3^ A W^−1^) were recorded over 75 h [80]. Solar‐driven electrochemical dissociation of H_2_S to H_2_ and sulfur products in photovoltaic‐electrochemical (PV‐EC) devices becomes an effective strategy for acid gas purification and energy‐saving H_2_ production. However, it suffers from several challenges. To address the inferior energy conversion efficiency and fussy multi‐step sulfur recovery problems, an integrated solar‐driven PV‐EC system with a diaphragm electrolytic reactor is proposed to solve these challenges. The optimized system integrated commercial silicon solar delivers a high solar‐to‐H_2_ energy conversion efficiency of up to 12 %, with approximately 99% H_2_ faradaic efficiency, and demonstrates at least 50 h of stability [42].

### 4.3 Electrocatalytic Conversion of H_2_S for H_2_

**FIGURE 5:**
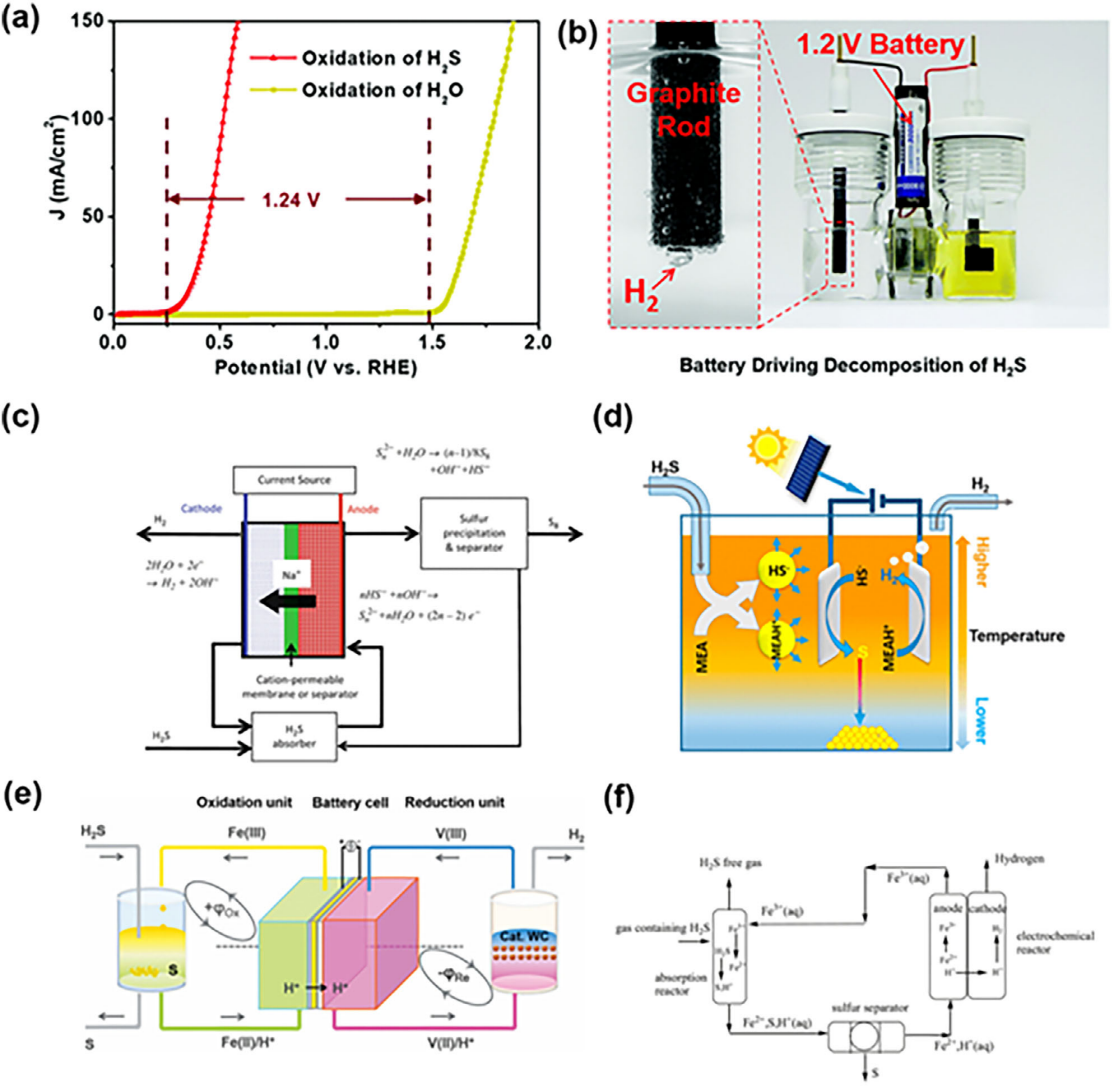
(a) Comparison of SOR and OER polarization curves for CoNi@NGs. The difference value of the two reactions’ onset potentials is 1.24 V. (b) The photo of a device with a 1.2 V commercial battery directly driving the decomposition of H_2_S. The enlargement highlighted in red shows the production of abundant H_2_ bubbles on the graphite rod [81]. Copyright 2020, Royal Society of Chemistry (c) Electrochemical process for sulfide oxidation coupled to H_2_ production [94]. Copyright 2014, Springer Nature (d) Conceptual design of H_2_S continuous electrolysis using organic solvents [95]. Copyright 2020, American Chemical Society. (e) Schematic illustration of the OFEC technology in acidic medium [96]. Copyright 2024, American Chemical Society. (f) Schematic diagram of the recovery of H_2_ from indirect electrochemical H_2_S splitting [97]. Copyright 2019, Elsevier.

Electrochemical methods for transforming H_2_S into H_2_ represent a promising area of research with significant potential for sustainable energy solutions. A key advantage is the low onset potential for H_2_S splitting, theoretically as low as 0.14 V, which is significantly lower than 1.23 V required for water splitting. In fact, Zhang et al. observed a 1.24 V difference in onset potentials when comparing SOR and OER (Figure 5a) [81]. During electrolysis, H_2_S breaks down, producing both elemental sulfur and H_2_ under applied potential on the electrode surface. At the anode, the H_2_S undergoes oxidation, yielding sulfur and protons. At the cathode, protons are reduced to form H_2_ gas. While the 0.14 V potential has not been widely achieved in practical applications, catalysts like Fe_2_NiSe_4_ have demonstrated electrocatalytic H_2_S splitting at a low overpotential of 440 mV, achieving a high current density of 100 mA cm^−2^ with 98% faradaic efficiency [41]. This reduced energy requirement enhances the sustainability of H_2_ production and improves its cost‐competitiveness. The process can be categorized into two primary approaches: direct and indirect electrolysis. While each method offers distinct advantages and faces specific technical challenges regarding sulfur extraction and reaction management (Summarized in Table 3), a significant hurdle remains: sulfur poisoning. This phenomenon is addressed in detail below, followed by an analysis of direct and indirect electrocatalytic systems.

**TABLE 3:** Comparison between direct and indirect electrolysis.

Feature	Direct electrochemical splitting	Indirect electrochemical splitting
Pathway	H_2_S → H_2_ + S (electrochemical)	H_2_S → Intermediate → H_2_ + S (chemical + electrochemical)
System Complexity	Simpler	More complex
Sulfur Management	More difficult (fouling, passivation)	Easier (separate sulfur loop)
Electrode Fouling Risk	High	Lower (especially at the anode)
Catalyst Stability	Limited	Potentially improved
Faradaic Efficiency	Moderate to High (if optimized)	Can be higher in controlled systems
Scalability	Challenging due to sulfur issues	More scalable in modular systems
Examples	MoS_2_/C, Ni‐foam, Ag/S cathodes	Polysulfide redox flow cells, S‐assisted water electrolysis
Energy Demand	Moderate	Moderate to Low (due to assisted oxidation)

#### 4.3.1 Sulfur Poisoning

Before evaluating specific catalytic approaches, the phenomenon of sulfur poisoning must be addressed. This process leads to rapid catalyst deactivation and remains the primary bottleneck in developing efficient SOR electrocatalysts. Due to the high electrical resistivity of elemental sulfur (≈1 × 10^15^ m Ω), its deposition on the electrode surface physically blocks active sites. This reduction in the electrochemically active surface area (ECSA), subsequently increase the anodic overpotential and degrades overall cell performance [21, 82]. The reaction mechanism of the SOR is heavily dictated by the chemical solubility of sulfur in the electrolyte. In acidic media, sulfur is largely insoluble; any precipitated sulfur quickly clogs the electrode pores and passivates the surface. Conversely, sulfur exhibits high solubility in alkaline media, where it reacts to form soluble polysulfides ($S_{n}^{2-})$ [83] Consequently, the majority of SOR studies, particularly those focusing on direct electrocatalysis, are conducted in alkaline conditions.

##### 4.3.1.1 Reaction Mechanisms

In alkaline environments, the primary oxidation mechanism is generally proposed as follows (Equations (15), (16), (17), (18), (19), (20), (21), (22), (23)): [83, 84, 85, 86]

(15) $$HS^{-}+OH^{-}\;\to S+H_{2}O+2e^{-}$$

(16) $$S^{2-}\to S+2e^{-}$$

The sulfur species can be directly oxidized to polysulfides or react with existing dissolved sulfur to form longer‐chain polysulfides:

(17) $$2HS^{-}+2OH^{-}\to {\;S}_{2}^{2-}+2H_{2}O+2e^{-}$$

(18) $$S+\;HS^{-}+OH^{-}\to S_{2}^{2-}+H_{2}O$$

(19) $$S+S^{2-}\to {\;S}_{2}^{2-}$$

In highly alkaline solutions, the oxidation process may proceed beyond polysulfides to include oxygenated species such as sulfite, thiosulfate, and sulfate. These side reactions reduce the Faradaic efficiency toward the desired elemental sulfur or polysulfide products:

(20) $$2HS^{-}+8OH^{-}\to S_{2}O_{3}^{2-}+5H_{2}O+8e^{-}$$

(21) $$S^{2-}+6OH^{-}\to {\mathrm{SO}}_{3}^{2-}+3H_{2}O+6e^{-}$$

(22) $$S_{n}^{2-}+6OH^{-}\to S_{2}O_{3}^{2-}+3H_{2}O+\left(n-2\right)S+6e^{-};n\;=2\;\mathrm{to}\;5$$

(23) $$S+4H_{2}O\;\to {\mathrm{SO}}_{4}^{2-}+8H^{+}+6e^{-}$$

The performance of SOR catalysts is governed by the adsorption energy of sulfide ions and intermediate sulfur species. According to the Sabatier principle, if the binding energy is too weak, the initial adsorption step limits the reaction rate. However, if the adsorption is too strong, the desorption of sulfur products is restricted. This strong adsorption leads to sulfur aggregation on the electrode surface, causing passivation. An ideal catalytic system must balance these interactions to prevent passivation while simultaneously providing high selectivity, stopping the reaction at the elemental sulfur/polysulfides rather than proceeding to deep oxidation (sulfates) [21, 87].

##### 4.3.1.2 Strategies to Mitigate Sulfur Poisoning

Strategies to mitigate catalyst poisoning focus on two primary objectives: minimizing sulfur accumulation on active sites and maximizing the solubility of the resulting products. These strategies can be categorized into (a) electrocatalyst design, (b) electrolyte modification, and (c) indirect electrolysis. While electrolyte selection and indirect methods are detailed in Sections 4.3.2 and 4.3.3, the next paragraphs focus on the electrocatalyst design. The primary goal of modern electrocatalyst design for the SOR is to create sulfiphobic surfaces that inherently resist sulfur adhesion. This is achieved by tuning surface wettability and contact angle, crystallographic face engineering, and electronic modulation.

###### 4.3.1.2.1 Surface Wettability and Contact Angle

The interaction between the solid catalyst and the liquid sulfur can be correlated to the contact angle (θ). Zhang et al. [88] investigated sulfur droplet behavior on various substrates at 120°C, identifying a critical threshold for passivation resistance. They found that substrates with a contact angle < 90° (such as carbon, θ = 29°) are sulfiphilic, making them prone to sulfur passivation. Conversely, substrates with θ>90° are sulfiphobic. Notably, NiS_2_ exhibited a contact angle of 108°, correlating with significantly improved resistance to sulfur poisoning compared to other sulfiphilic catalysts, such as Pt, Ni, and Pt/MMo.

###### 4.3.1.2.2 Crystallographic Face Engineering

Surface orientation plays a decisive role in determining binding affinity. An early study by Tang [89] demonstrated that in Copper (Cu) catalysts, the (110) plane is more susceptible to sulfur passivation than the (111) plane. More recently, Xiao et al. [90] utilized DFT calculations to study Co_3_S_4_ nanowires, revealing that the (311) facet possesses a remarkably low energy barrier (0.09 eV) for sulfur desorption. These findings suggest that tailoring the exposed facets of a nanocatalyst can enable a “self‐cleaning” mechanism by facilitating rapid sulfur release.

###### 4.3.1.2.3 Electronic Modulation and HSAB Theory

The d‐band center position is a key descriptor for sulfur adsorption. A downward shift of the d‐band center (away from the Fermi level) weakens the metal‐sulfur bond, facilitating intermediate desorption. This electronic relationship can also be explained through Hard‐Soft‐Acid‐Base (HSAB) theory [91]. Pei et al. [92] demonstrated this principle using CuCoNiMnCrS_x_ high‐entropy sulfide catalysts, which demonstrated electrocatalytic H_2_S splitting at a low overpotential of 250 mV for SOR, achieving a high current density of 100 mA cm^−2^ with 96% faradaic efficiency. They noted that soft‐soft combinations, such as the covalent bond between soft‐acid (Cu^+^) and soft‐base (S^2−^), create high orbital overlap between sulfur p‐orbitals and copper d‐orbitals, leading to irreversible poisoning. Therefore, by doping the lattice with harder‐acid high‐valent cations (M), they created an asymmetric Cu‐S‐M structure. This soft‐hard combination induces a repulsion‐attraction effect that increases the electron cloud density on Cu sites, stabilizing the Cu^+^ state under oxidation conditions. This electronic asymmetry prevents the formation of overly strong covalent bonds, thereby promoting the continuous adsorption and oxidation of sulfide ions without surface passivation.

Apart from sulphur poisoning, the pH of the electrolyte plays an important role; its value, along with the specific electrocatalyst used, significantly influences the overall reaction mechanism and efficiency. In this line, different electrolytes have been used, and here we discuss them in detail in the following section, including acidic, alkaline, organic, and ionic liquids.

#### 4.3.2 Direct Electrolysis

In this approach, an electric potential is applied to an aqueous solution containing dissolved H_2_S. At the anode, H_2_S is oxidized to produce elemental sulfur and protons, while at the cathode, these protons are reduced to generate H_2_ gas. The overall process reaction can be summarized as follows (Equations (24), (25)):

Anode Reaction:

(24) $$H_{2}S\to S+2H^{+}+2e^{-}$$

Cathode Reaction:

(25) $$2H^{+}+2e^{-}\to H_{2}$$

This method is energy‐efficient because the required cell potential closely aligns with the difference between the hydrogen evolution reaction (HER) and SOR, allowing for lower operating voltages. However, a major challenge with direct electrolysis is the formation of elemental sulfur on the electrocatalyst surface, which can lead to electrode passivation and reduced system efficiency. A digital image of a typical cell and a schematic diagram is shown in Figure 5b [81]. Additionally, the in situ separation of sulfur from the electrolyte during the reaction is technically difficult, posing challenges for continuous operation and scalability. To prevent this, the degree of oxidation must be carefully regulated. A serious concern is also the diffusion of sulfide species through the membrane to the cathode, as these species can irreversibly poison most of the cathode materials [93]. This poisoning diminishes their catalytic activity and shortens their operational lifespan. Finally, the pH of the electrolyte plays an important role; its value, along with the specific electrocatalyst used, significantly influences the overall reaction mechanism and efficiency. In this line, different electrolytes have been used, and here we discuss them in detail in the following section, including acidic, alkaline, organic, and ionic liquids.

##### 4.3.2.1 Alkaline Medium

Alkaline electrolytes such as KOH or NaOH enhance the conductivity of the system, improve electrode stability, and facilitate the separation of H_2_ and sulfur species [84]. Moreover, the electrochemical potential required to oxidize sulfide to elemental sulfur or polysulfides is significantly lower than that required for water oxidation, allowing for low‐energy H_2_ production when coupled with HER at the cathode. In 1990, a study by Kautek et al. demonstrated the electrochemical decomposition of H_2_S into high‐purity H_2_ and crystalline elemental sulfur using a double‐compartment cell with a Nafion membrane separator [98]. Electrolysis was conducted at 80°C in alkaline solutions containing equimolar NaOH and NaHS, as well as Na_2_S and Na_2_S_4_ electrolytes. Under optimized conditions, without continuous H_2_S bubbling, anodic passivation by sulfur and undesirable side reactions such as oxygen evolution and sulfur oxyanion formation were effectively suppressed. In a study by Yang et al., a reactor, depicted in Figure 5c, was employed to selectively oxidize hydrogen sulfide (HS^−^) ions at the anode while simultaneously facilitating H_2_ evolution at the cathode [99]. This configuration operates with a reactor equilibrium potential difference (ΔU) that is significantly lower than what is typically required when oxygen evolution is the anodic reaction. As a result, it enables more energy‐efficient operation. Under optimized conditions, the system demonstrated the co‐production of high‐purity H_2_ and elemental sulfur at an impressive current density of 3 kA m^−2^, with a corresponding reactor potential difference of approximately 1.0 V [94]. This electrolyzer operates at moderately elevated temperatures in the range of 80°C–90°C. These elevated temperatures not only help lower the overall cell voltage required for operation but also reduce the likelihood of forming an insulating layer of elemental sulfur on the anode surface. Such sulfur deposition, if uncontrolled, can severely hinder electrode activity by passivating the anode, ultimately decreasing system performance. To mitigate this issue, precise control over both the anode potential and the electrolyte composition is essential. This approach helps maintain favorable reaction pathways and minimizes the formation of undesirable byproducts like solid sulfur films. However, in cases where sulfur deposition does occur, the anode, particularly when composed of carbon fiber materials, can often be regenerated by applying a reductive potential [100]. This reduction process strips the sulfur layer from the electrode surface, thereby restoring its electrochemical activity and enabling continued operation without the need for electrode replacement.

Yang et al. studied that sulfur formation proceeded primarily via oxidation of HS^−^, S^2−^, and polysulfides, all occurring at similar anodic potentials [101]. Current efficiencies for H_2_ and sulfur production reached ≥95%. The process was conducted in batch mode, though a continuous flow system was proposed for scalability. Various anode materials (graphite, nickel, nickel‐chromium alloy, titanium) and cathodes (nickel or graphite) were tested. The electrochemical performance was evaluated in two sulfide‐containing electrolytes: Na_2_HPO_4_ (pH 9.2) and NaOH (pH 13). At pH 9.2, significant sulfur deposition on the Ni surface, ranging from dense S_8_ films to porous sulfur structures, led to reduced electrochemical activity and partial conversion of Ni to NiS_2_. In contrast, operating at pH 13 in NaOH (−1.0 V to +0.6 V vs. Hg/HgO) suppressed sulfur passivation by stabilizing sulfur in the form of soluble polysulfide species (e.g., S_2_^2−^, S_8_^2−^). These findings confirmed the feasibility of direct H_2_S electrolysis in alkaline media by careful control of pH, temperature, and electrolyte composition, establishing a basis for sustainable H_2_ and sulfur co‐production. Zhang et al. developed a graphene‐encapsulated, nitrogen‐doped CoNi nanoalloy (CoNi@NGs) catalyst as a non‐precious anode material for efficient electrocatalytic H_2_ production from H_2_S [81]. Operating in Ar‐saturated 1 M NaOH and 1 M Na_2_S electrolytes, the catalyst achieved an onset potential of 0.25 V, 1.24 V lower than the water oxidation potential and delivered nearly twice the current density of Pt/C (Figure 5a). It exhibited ∼98% H_2_ Faradaic efficiency and exceptional long‐term durability exceeding 500 h without performance decay. DFT studies confirmed that CoNi alloying and nitrogen doping synergistically promoted polysulfide formation on graphene surfaces. This demonstration system achieved 1200 h of stable operation in H_2_S‐contaminated industrial syngas, highlighting the catalyst's promise for sustainable H_2_ generation from sulfur‐containing feedstocks.

In a similar study, a special nickel foam electrode wrapped in two layers of graphene called Ni@NC foam has shown excellent results for splitting H_2_S, where the first graphene layer protected nickel and improved electron flow, while the second layer, made of graphene‐coated nickel nanoparticles on carbon fibers, increased the number of active sites for the reaction. The study was conducted in 1 M NaOH and 1 M Na_2_S, where the electrode reached over 1 A/cm^2^ at just 1.12 V, which is five times better than regular nickel foam, and stayed stable for more than 300 h. In a demo system removing H_2_S from natural gas, it completely removed 20% of H_2_S, produced 95% efficiency, and used 43% less electricity than traditional water electrolysis, making it a viable option for clean H_2_ production and H_2_S removal [102].

Another study by Kumar et al. employed a mixed‐phase (1T/2H) NiCu‐MoS_2_ micro‐flower catalyst that was developed for direct H_2_S electrolysis, enabling simultaneous anodic SOR and cathodic H_2_ production in 1 M NaOH saturated with H_2_S [103]. The catalyst exhibited a low onset potential for SOR (0.21 V vs. RHE), which is 1.02 V lower than the thermodynamic potential for OER, and maintained a high H_2_ Faradaic efficiency of 98.1% over 150 h. When assembled into a two‐electrode H_2_S electrolyzer, the system achieved efficient H_2_ generation at a remarkably low cell voltage of 0.7 V, well below the requirements for conventional water splitting. All electrochemical evaluations were performed using a graphite paper‐supported working electrode and a standard three‐electrode setup. This work demonstrates a practical and energy‐efficient strategy for valorizing H_2_S through direct splitting into pure H_2_ and elemental sulfur. Another study by Kumar et al. incorporated a nanorod‐embedded CoFeS_2_ catalyst integrated with a nitrogen‐doped carbon framework for direct H_2_S electrolysis, achieving simultaneous cathodic HER and anodic SOR in 1 M NaOH saturated with H_2_S [104]. The catalyst demonstrated a low onset potential of 0.23 V (vs. RHE), approximately 1.0 V below the thermodynamic potential of water splitting, and sustained a high H_2_ Faradaic efficiency of 97.8% over 120 h. In recent years, metal selenides and nanostructured materials have garnered significant attention for their exceptional electrochemical performance. A study by Ding et al. explored a robust array of Fe_2_NiSe_4_ nanowires grown in situ on FeNi_3_ foam (Fe_2_NiSe_4_/FeNi_3_) as a bifunctional electrocatalyst for ambient‐condition electrochemical H_2_S splitting [41]. Synthesized via hydrothermal treatment, the nanowire array provides abundant active sites and enhanced electron transport pathways, facilitating both SOR and HER. In a standard three‐electrode configuration using 1.0 M NaOH with 1.0 M Na_2_S as the electrolyte, the Fe_2_NiSe_4_/FeNi_3_ electrode achieved a current density of 100 mA cm^−2^ at a low cell potential of 440 mV, with ∼98% Faradaic efficiency for H_2_ production. These results underscore the material's potential for efficient desulfurization and sustainable H_2_ generation under mild conditions.

The development of a multiwalled carbon nanotube‐graphene oxide (MWCNT/GO) nanocomposite, which leverages synergistic effects of structural integration and heteroatom doping (N, S, and O) to enhance electrochemical activity toward H_2_S oxidation, was studied by Narwade et al. [105]. This hybrid catalyst achieved high current densities approaching 98 mA cm^−2^ at relatively low onset potentials (−0.5 V vs. SCE), highlighting its potential as a metal‐free alternative to conventional transition‐metal catalysts. The incorporation of graphene oxide provides abundant defect sites and improved electron transport, while the tubular MWCNT architecture promotes high conductivity and structural robustness.

Electrochemical H_2_S splitting in alkaline media has been more extensively investigated; however, catalyst passivation remains a major challenge that limits long‐term operation, highlighting the need for further research in this area.

##### 4.3.2.2 Organic Solvents and Ionic Liquids

To mitigate sulfur poisoning, strategies such as the addition of organic solvents to the electrolyte and the use of ionic liquids have been investigated. The use of organic solvents to address this issue was first reported in 1986 by Shih et al. [106]. They examined sulfur extraction during the electrochemical oxidation of sulfide solutions by introducing solvents such as toluene or benzene into the H_2_S electrolysis system. These solvents dissolved the sulfur deposited on the anode surface, enabling continuous removal of the product mixture from the cell and preventing electrode passivation. Under conditions of 60°C and 0.06 M sulfide concentration, this approach achieved a conversion rate of up to 60% of sulfide ions to sulfur. However, maintaining optimal electrolyte conditions, managing the accumulation of elemental sulfur, and scaling the process for industrial use pose significant logistical challenges.

More recently, in 2020, Ma et al. reported an alternative approach for simultaneous recovery of H_2_ and sulfur via H_2_S electrolysis using a medium containing tetraethylene glycol dimethyl ether as the solvent, the ionic liquid [C_3_OHmim]BF_4_ as the supporting electrolyte, and monoethanolamine (MEA) as the H_2_S absorbent [95]. This system successfully addressed catalyst passivation issues typically caused by sulfur poisoning, while also demonstrating a more straightforward and faster route for solvent recovery (Figure 5d).

As stated earlier, changes in pH significantly influence the reactivity of H_2_S. In alkaline conditions, H_2_S readily dissociates into HS^−^ and S^2−^ ions, increasing the concentration of reactive species that interact strongly with the electrode surface and promote faster oxidation. In contrast, in acidic media, H_2_S mainly remains in its neutral molecular form, which adsorbs weakly on the electrode and results in slower direct oxidation kinetics. Despite the energy‐efficiency advantages of direct electrochemical H_2_S splitting, major operational challenges remain, particularly catalyst poisoning and sulfur deposition that reduce activity and shorten electrode lifespan. Acidic systems have proven especially difficult to manage, prompting most research to concentrate on alkaline electrolytes. However, newer approaches involving organic solvents and ionic liquids are emerging as alternative strategies to improve stability and performance. Continued innovation in catalyst design, electrolyte engineering, and system integration is crucial to overcoming these barriers and enabling large‐scale industrial implementation of H_2_S electrolysis [22, 107]. A summary of electrocatalysts that have been used, and their electrolyte, is provided in Table 4.

**TABLE 4:** Performance comparison of catalysts for direct electrochemical H_2_S splitting.

Catalyst	Electrolyte	H_2_S splitting onset potential for SOR @j	Overall faradaic efficiency	References
CoNi nanoparticles encapsulated in graphene shells	1 M NaOH and 1 M Na_2_S	0.25 V (vs RHE) at 1 mA/cm^2^	98%	[81]
Mixed phase NiCu‐MoS_2_	H_2_S saturated 1 M NaOH	0.21 V (vs RHE) at 10 mA/cm^2^	98.1%	[103]
CoFeS_2_	H_2_S purged in 1 M NaOH	0.23 V (vs RHE) at 10 mA/cm^2^	97.8%	[104]
Fe_2_NiSe_4_ nanowires array	1 M NaOH with 1 M Na_2_S	0.44 V (vs RHE) at 100 mA/cm^2^	98%	[41]
Ni@NC Foam	1 M NaOH with 1 M Na_2_S	1.12 V (vs RHE) at 1 A/cm^2^	95%	[102]
Graphite Sheet	H_2_S purged in 1 M H_2_SO_4_	1.05 V (vs RHE)	∼100%	[96]
Multiwalled Carbon nanotube‐ graphene oxide nanocomposite	H_2_S purged in 0.5 M KOH	−0.5 V (vs SCE) at 1 mA/cm^2^	Not reported	[105]
NiSe nanowire array catalyst	1 M NaOH + 1 M Na_2_S	0.4 V (vs RHE) at 30 mA/cm^2^	∼99%	[108]
Cu_2_S/Ni Foam Catalyst	1 M NaOH + 1 M Na_2_S	0.26 V (vs RHE) at 10 mA/cm^2^	∼86%	[109]
WS_2_ nanosheets	1 M NaOH + 1 M Na_2_S	0.45 V (vs RHE) at 10 mA/cm^2^	∼99.22%	[110]
Ni_3_S_2_/Ni_3_N	H_2_S purged in 1 M NaOH	0.25 V (vs RHE) at 10 mA/cm^2^	>95%	[111]
Pt	H_2_S purged in Tetraethylene glucol dimethyl ether, ionic liquid [C_3_OHmim]BF_4_ and MEA	−0.5 V (vs Ag/Ag^+^) at 200 mA/cm^2^	89%	[95]

#### 4.3.3 Indirect Electrolysis

To reduce electrode passivation, indirect electrolysis has been implemented, which besides separating anodic and cathodic counterparts, it also contains a second reactor that separates or absorb the species of interests. Indirect electrolysis avoids sulfur poisoning. For example, D.W. Kalina, et al., produced H_2_ gas where H_2_S was converted to H_2_ and sulfur in a strong alkaline Iodide solution [112]. Electrochemically generated Iodine then yields iodate, which then reacts with H_2_S to yield sulfur. The electrochemical reactions are shown below (Equations (26), (27), (28), (29)):

Anode Reaction:

(26) $$3I^{-}\to {I_{3}}^{-}+2e^{-}$$

Cathode Reaction:

(27) $$2H^{+}+2e^{-}\to H_{2}$$

Overall Reaction:

(28) $$3I^{-}+2H^{+}\to {I_{3}}^{-}+H_{2}$$

Reaction in chamber:

(29) $$I{O_{3}}^{-}+3H_{2}S\to 3S+3H_{2}O+I^{-}$$

The reaction between H_2_S and IO_3_ proceeds efficiently, yielding a microcrystalline precipitate that can be extracted into a water‐immiscible organic solvent like toluene. Recrystallization from toluene produces a sulfur product with low iodine contamination, measuring less than 30 ppm. Iodine losses from the anolyte due to volatilization are minimal. However, the overall sulfur yield remains relatively low at 77% based on the initial H_2_S input. Consequently, while current efficiencies are high, the overall reaction efficiency is limited. Although the direct method is efficient and simple, it has a lower sulfur yield and relies on the use of organic solvents. The electrolysis method can be optimized with electrocatalysts for better efficiency and offers greater long‐term potential by providing both H_2_ and S as valuable products, making it a more versatile and environmentally friendly approach. One of the trade‐offs for indirect reduction is that the overall process often requires a higher voltage to oxidize the intermediate, which can reduce energy efficiency. Furthermore, some intermediates may degrade over time or migrate across the membrane, potentially leading to system instability or loss of activity. Hence, optimizing both membrane selectivity and reaction conditions is critical to minimizing cross‐contamination and ensuring efficient, long‐term electrochemical cell performance.

An acidic medium is not common for electrochemical H_2_S splitting because it can seriously degrade the catalysts. However, a recent study by Wang et al. proposed an innovative electron‐mediated off‐field electrocatalysis (OFEC) approach for the complete splitting of H_2_S into H_2_ and elemental sulfur under ambient conditions using 1 M H_2_SO_4_ electrolyte (Figure 5e) [96]. The process employs Fe(III)/Fe(II) and V(II)/V(III) redox couples to decouple H_2_S oxidation and proton reduction. Fe(III) oxidizes H_2_S with nearly 100% conversion to sulfur, while V(II) reduces protons to H_2_ on a non‐precious tungsten carbide catalyst. These mediators are regenerated in an external electrolyzer operating at a low cell voltage of 1.05 V, close to the theoretical minimum of 1.02 V. The system achieves an energy consumption of 2.8 kWh/Nm^3^ H_2_ using Fe and V redox pairs, which can be further reduced to 0.5 kWh/Nm^3^ H_2_ by replacing them with heteropolyacid/quinone mediators. This OFEC strategy not only eliminates H_2_S emissions and recovers valuable H_2_ but also offers a scalable, cost‐effective, and energy‐efficient solution for industrial gas treatment and electrochemical reactions involving solid products. Nevertheless, electrochemical H_2_S splitting in acidic media remains very challenging, which is why most of the research on this topic focuses on alkaline medium.

In a recent study, H. Huang et al. demonstrated H_2_ recovery from H_2_S using Fe^3+^/ Fe^2+^ as an electrochemical intermediate in acidic media. The indirect electrochemical process employed coupled absorption and electrolysis reactors (Figure 5f), enabling simultaneous recovery of H_2_ and sulfur while preventing catalyst poisoning by removing sulfur prior to the electrocatalytic step. Although indirect H_2_S electrolysis remains relatively unexplored, it represents a promising alternative pathway for H_2_ recovery from H_2_S [97].

### 4.4 Plasma‐Assisted Conversion of H_2_S for H_2_

Conventional H_2_S conversion via the Claus process recovers sulfur but wastes H_2_ as water, while thermo‐catalytic conversion faces thermodynamic and energy barriers. Photo and electrocatalytic conversion of H_2_S to H_2_ also suffers from catalyst corrosion. Plasma‐assisted technologies have emerged as a promising alternative for H_2_S conversion into H_2_ and elemental sulphur. In this section, we will be focusing on reaction mechanisms, reactor engineering, catalyst integration, and scalability challenges associated with the plasma‐assisted conversion approach. By integrating plasma and catalysis, sustainable and scalable H_2_ production from H_2_S streams, aligning with circular economy principles, can be achieved [113, 114]. Plasma is called the fourth state of matter, and it is the most abundant form of matter in the universe, consisting of ionized gas with species like ions, electrons, and free radicals. Plasma also naturally exists in the form of auroras, solar wind, the sun's corona, and the ionosphere. Plasma can also be artificially produced by exciting neutral gas with external energy. Its generation can be achieved through thermal excitation, which involves heating the gas to very high temperatures initiating ionization; however, this method is rarely used due to high energy demands. Usually, plasma is generated by electrical excitation, where high voltage is applied to the gas that energizes electrons, initiating collisions that form plasma species by ionization, excitation, and dissociation of gases [23]. Plasma can be generally categorized into thermal and non‐thermal types [48, 115, 116]. Thermal plasmas can reach temperatures of several thousand degree celsius that makes them suitable for high‐temperature applications such as chemical synthesis, metallurgical processes, medical waste treatment, and general waste management. On the other hand, non‐thermal plasmas, known as cold plasmas, can work at ambient temperatures and can be utilized for low‐temperature applications like sterilization, food processing, decontamination, and carbon conversion [117, 118, 119, 120, 121]. In plasma‐based H_2_S conversion, thermal and non‐thermal plasma can be used efficiently. In the plasma process, H_2_S feed is exposed to a plasma environment, and energized plasma species such as electrons and radicals interact with H_2_S, breaking it into various independent species that form new products through subsequent interactions and reactions. This approach significantly reduces temperature and pressure requirements from above 1000°C and high pressure to room temperature and atmospheric pressure [122]. The feasibility and efficiency of the plasma‐based H_2_S conversion process depend on factors such as plasma source, feed composition, plasma catalyst, and retention time, with modifications to these factors affecting energy input, conversion, yield, and selectivity [123, 124, 125, 126, 127, 128]. Non‐thermal plasma is characterized by the presence of highly energetic electrons (1–10 eV) in a low‐temperature bulk gas. These electrons drive chemical transformations by colliding with gas‐phase molecules, leading to excitation, ionization, and dissociation without the need for high bulk temperatures. In the process of H_2_S decomposition, non‐thermal plasma facilitates the activation of H_2_S molecules at near‐ambient conditions, overcoming the thermodynamic limitations that constrain conventional thermal and catalytic processes. The conversion of H_2_S in plasma environments proceeds via a combination of electron impact dissociation and radical‐mediated reactions (Equations (30), (31), (32), (33), (34)). The major steps can be summarized as follows:

Electron Impact Dissociation:

(30) $$H_{2}S+e^{-}\to \mathrm{HS}\cdot +H\cdot +e^{-}$$

(31) $$\mathrm{HS}+e^{-}\to H\cdot +S+e^{-}$$

Radical Recombination:

(32) $$2H\cdot \to H_{2}$$

(33) $$2S\to S_{2}$$

The overall reaction:

(34) $$H_{2}S\to H_{2}+S_{2}$$

**FIGURE 6:**
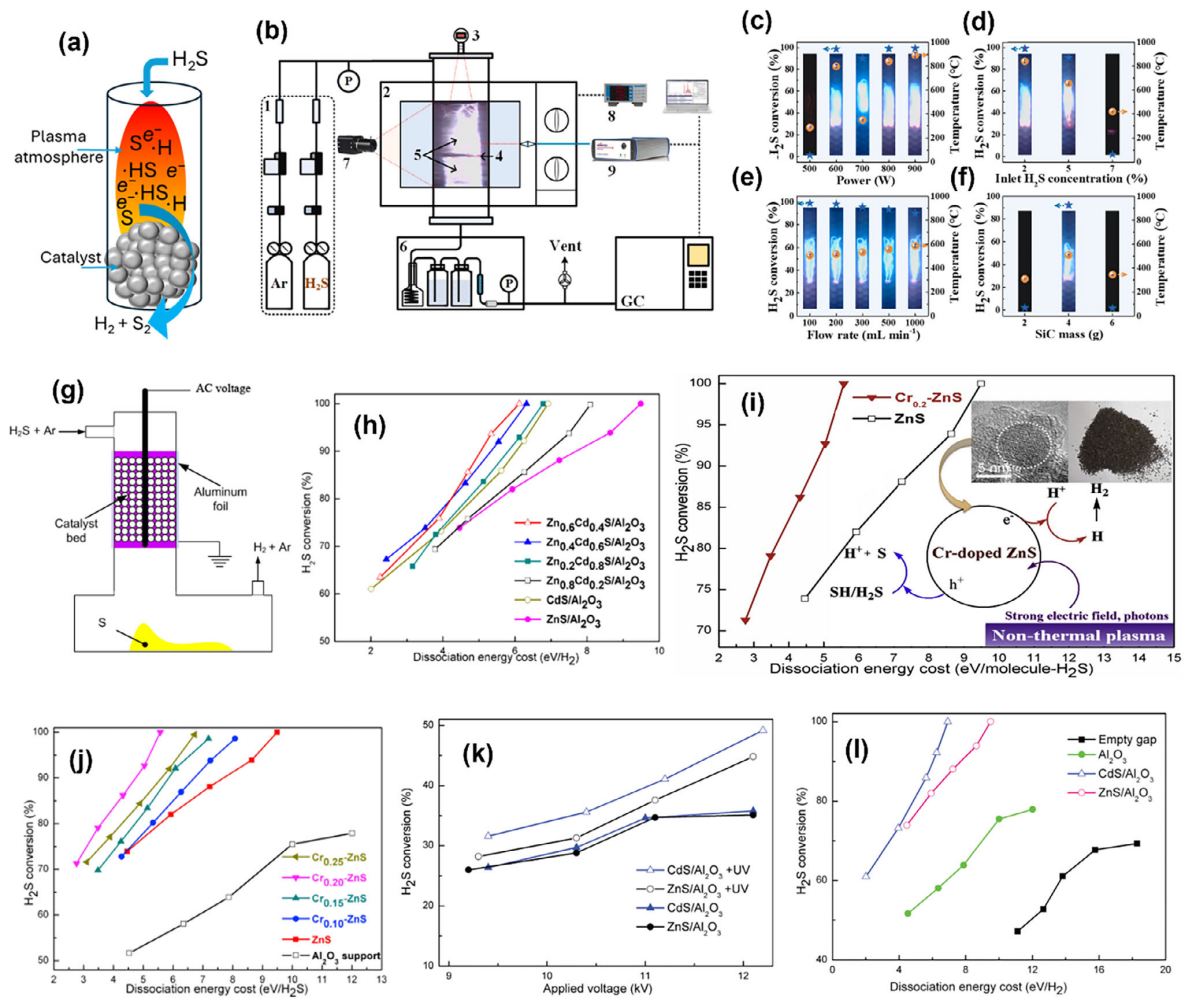
(a) Schematic illustration of catalytic H_2_S decomposition into H_2_ and S_2_/S_8_ in plasma atmosphere. (b) Schematic of the experimental setup: 1. Feed gas control unit; 2. Microwave plasma reactor unit; 3. Infrared thermometer; 4. SiC; 5. SiC‐mediated microwave‐induced discharge plasma; 6. Product condensation‐absorption unit; 7. Industrial camera; 8. Dynamometer; 9. Spectrometer. Effects of (c) microwave power, (d) inlet H_2_S concentration at 800 W, (e) gas flow rate, and (f) SiC mass for 5% H_2_S/Ar on H_2_S decomposition via microwave plasma [35]. Copyright 2024, Elsevier (g) Schematic diagram of the DBD reactor. (h) H_2_S conversion as a function of dissociation energy cost in the plasma‐induced decomposition over Zn_x_Cd_1‐x_S/Al_2_O_3_, CdS/Al_2_O_3_ and ZnS/Al_2_O_3_ catalysts [129]. Open Access Journal (i) Cr‐doped ZnS semiconductor catalyst with high catalytic activity for H_2_ production from H_2_S in non‐thermal plasma. (j) H_2_S conversion as a function of dissociation energy cost in the plasma‐induced decomposition over the Cr_x_‐ZnS catalysts with different Cr/Zn molar ratios [132]. Copyright 2019, Elsevier (k) H_2_S conversion as a function of dissociation energy cost in the plasma‐induced decomposition with and without porous pellets filled in the gap. (l) H_2_S conversion as a function of applied voltage (peak‐to‐peak voltage) of the generator when the downstream catalyst bed was with and without UV lamp irradiation [130]. Copyright 2019, Royal Society of Chemistry.

Here, reactive species like high‐energy electrons directly dissociate H_2_S via electron impact to H, HS, and S free radicals that later recombine to form H_2_ and S_2_ or S_8_. Unlike thermal decomposition, which is limited by equilibrium conversion (typically <40% at 900°C), plasma processes can achieve higher conversions at much lower bulk temperatures. This is due to the non‐equilibrium nature of plasma, where energetic electrons enable reactions that are otherwise kinetically or thermodynamically inaccessible. To make the process more efficient and selective, suitable catalysts can be used. Plasma catalysis combines the synergistic effect of plasma and catalysts to produce desired products at specific rates and enhanced efficiencies compared to traditional catalytic processes. Figure 6a shows the mechanism of H_2_S decomposition, where the electric field and photons split the feed and balance gas into ions, electrons, neutrals, excited molecules, and other plasma species. Multiple decomposition and recombination reactions occur simultaneously, as depicted in Figure 6a, yielding gaseous H_2_ and elemental sulfur. Plasma catalysts are getting attention due to the exclusive synergistic mechanism combining photon radiation with catalysts that is enhancing reactivity, selectivity, and efficiency of the process compared to conventional thermal catalytic approaches. Compared to the thermocatalytic process, the catalytic plasma method works with a small charge at ambient temperature, making them more sustainable alternative. Screening suitable plasma catalysts is crucial for high H_2_ yields. Figure 6b shows the experimental setup for H_2_S decomposition using SiC‐mediated microwave‐induced discharge plasma, which comprises four main units: feed gas control, microwave plasma reactor, condensation–absorption, and analytical detection. The multimode microwave plasma reactor was adapted from a 2.45 GHz Galanz commercial microwave oven by integrating a variable‐frequency power supply and a water‐cooled magnetron, enabling continuous and stable power delivery from 300 to 1100 W, monitored by a dynamometer. A heat‐resistant quartz tube (1 mm wall thickness, 20 mm outer diameter) runs vertically through the center of the multimode cavity, with cylindrical metal meshes installed at both openings to prevent microwave leakage. Inside the quartz tube, SiC is positioned on a porous sieve plate, and an infrared thermometer is aligned with the upper surface of the SiC to measure temperature. Figure 6c–f illustrates the influence of SiC position, microwave power, pressure, and Ar flow rate on plasma morphology and SiC's steady‐state temperature. In Figure 6c, plasma distribution varies with SiC position due to non‐uniform microwave field strength within the cavity. As shown in Figure 6d, plasma length increases as microwave power rises from 300 W to 600 W, then plateaus beyond 600 W because of cavity height constraints.

Notably, when plasma reaches the upper cavity wall, a discharge resembling dielectric barrier discharge appears at the quartz tube aperture, preventing plasma from extending outside the cavity. SiC temperature generally increases with microwave power, though the effect is less pronounced between 600–800 W due to heat dissipation. Although H_2_S decomposition was conducted at atmospheric pressure, pressure effects on plasma characteristics were also examined (Figure 6e). Finally, Figure 6f shows that higher Ar flow slightly shortens plasma length by suppressing reverse gas flow, while SiC temperature and discharge density rise, likely due to increased excitation of ground‐state Ar atoms [128]. Catalysts in the plasma provide surfaces that radically alter these reaction pathways and improve efficiency. There are several synergistic roles a catalyst can play in a plasma‐H_2_S system.

Metal sulfides and semiconductor catalysts (e.g. CdS, ZnS, MoS_2_) exhibit high activity and stability under plasma conditions. They often achieve the highest H_2_S conversions (90%–100%) and are resistant to sulfur poisoning since their active phase is already sulfided. These catalysts benefit from plasma excitation (as explained previously) and maintain performance over long runs. For example, Zhao et al. used a Dielectric Barrier Discharge (DBD) reactor, a schematic of which is shown in Figure 6g. They reported that Zn and Cd sulfides supported on Al_2_O_3_ achieved up to 100% conversion, which was maintained for 100 h. Furthermore, they found that Zn_0.6_Cd_0.4_S/Al_2_O_3_ achieved 100% conversion with the lowest specific dissociation energy cost (eV/H_2_), as detailed in Figure 6h [48, 129, 130, 131].

CoS supported on γ‐Al_2_O_3_ and doped with BaMn_0.2_Cu_0.8_O_3_ has demonstrated exceptional performance under MW‐plasma conditions. Operating at 788°C, this composite catalyst achieved an H_2_S conversion rate of 80.33%, significantly surpassing the equilibrium limit for thermal decomposition at similar temperatures. The enhanced activity is attributed to the synergistic interaction between CoS and the perovskite oxide, which together facilitate efficient microwave absorption and radical recombination. This system exemplifies how tailored catalyst architecture can leverage microwave energy to overcome thermodynamic constraints [133]. Building simple sulfides, researchers have improved performance by doping or combining catalysts. Zhao et al. [132]. Figure 6j illustrates the performance across different Cr doping concentrations, showing that all the doped catalysts demonstrated superior activity for H_2_S decomposition compared to undoped ZnS and Al_2_O_3_. Specifically, the Cr_0.20_‐ZnS composition exhibited the optimal performance, achieving 100% H_2_S conversion at the lowest energy cost (5.57 eV/H_2_S) and comparing it with ZnS (Figure 6i). Furthermore, characterization confirmed that the Cr^3+^ ions were uniformly dispersed within a cubic sphalerite structure, avoiding the formation of inactive impurity phases. This high efficiency is attributed to favorable structural properties: the catalysts possess high BET surface areas and small particle sizes (∼8 nm). These factors are critical as they maximize the number of active centers, enhance light absorption, and accelerate the transport of photo‐generated carriers, effectively preventing electron‐hole recombination and boosting overall catalytic activity. This highlights how tuning the electronic properties of catalysts can significantly enhance plasma‐catalytic efficiency. Similarly, composite catalysts, such as Mo_2_C‐Co_2_C on SiC (derived from a carbonized molybdenum/cobalt catalyst), have demonstrated a conversion of ∼90% for H_2_S in microwave plasmas [134]. Combining metals can provide multiple active sites; in this case, Mo and Co carbides likely facilitated H‐S bond scission and H_2_ desorption, respectively.

In another study, Zhao et al. also reported that plasma‐generated H and HS radicals can adsorb on catalyst surfaces and recombine to form H_2_ and surface‐bound sulfur more selectively. This removes radicals from the gas phase, preventing back‐reactions, and catalyzes the formation of H_2_. This effect is demonstrated by packing an inert porous material (e.g. Al_2_O_3_) in a DBD reactor, which not only changes the discharge characteristics but also provides surface area for radicals to stick, increasing their residence time. Figure 6k shows that simply adding Al_2_O_3_, CdS‐Al_2_O_3_, or ZnS‐Al_2_O_3_ pellets in a DBD gap raised H_2_S conversion from ∼67% to ∼75% and reduced the specific energy cost for H_2_. Figure 6l compares H_2_S conversion as a function of applied voltage of the generator in the presence of CdS‐Al_2_O_3_ and ZnS‐Al_2_O_3_, testing the impact of UV irradiation. Without UV irradiation, the overall conversion for CdS–Al_2_O_3_ was comparable to that for ZnS‐Al_2_O_3_, indicating that the catalysts in the downstream might not contribute to the decomposition. When the UV lamp was on, an enhancement of H_2_S decomposition was observed for both CdS‐Al_2_O_3_ and ZnS‐Al_2_O_3_. It is evident that photocatalysis by these composites enhances the decomposition of H_2_S when irradiated with UV light [130].

Therefore, plasma‐based decomposition of H_2_S is a promising approach for H_2_ production and sulfur recovery, holding the potential to complement or even replace conventional processes in certain contexts. Although significant progress has been made, understanding reaction mechanisms, optimizing reactor designs, energy efficiency, scalability, and sulfur management remain fundamental challenges. Further research is still needed in both academia and industry, particularly in areas such as reactor design, process intensification, catalyst durability, and system integration. These subjects remain critical for realizing the full potential of plasma‐driven H_2_S conversion for a sustainable H_2_ economy.

## 5 Impact and Techno‐Economic Viability

Technology validation is essential to move from lab‐scale proof‐of‐concept to pilot‐scale demonstration. Techno‐economic analyses indicate that H_2_S splitting could be competitive with traditional H_2_ production if integrated with sulfur recovery units and renewable energy inputs [135]. A key barrier for low‐carbon H_2_ is the cost gap with H_2_ from fossil fuels. At present, producing H_2_ from fossil fuels is the most inexpensive option in most areas of the world. Depending on regional gas prices, the H_2_ production cost from natural gas usually ranges from 0.5 to 1.7 USD/kilogram (kg). But it comes with high CO_2_ emission. A multiscale techno‐economic analysis of H_2_ production from H_2_S‐based process reported H_2_ production costs of about 2.23 USD/kg at ∼600 kg H_2_/h scale, in favorable sour‑gas compositions and high sulfur recovery [136]. The thermodynamic dissociation energy of H_2_S is lower than that of water, but in real practice, it could require higher energy for heating, plasma power, or electrochemical overpotentials. However, environmental benefits, including reduced SO_2_ emissions and circular use of industrial waste gases, further justify the ongoing research interest. Policy support and scale‐up demonstration are necessary for real‐world adoption. The splitting of H_2_S into H_2_ and elemental sulfur is gaining interest as a promising approach to waste valorization. Continued progress hinges on breakthroughs in materials science, reactor engineering, and system integration.

Thermal and plasma‐based methods have high energy demands due to the endothermic nature of H_2_S decomposition. Electrochemical systems, while potentially scalable, currently require expensive catalyst materials. Moreover, process integration would be an effective initiative to achieve high efficiency. For example, membrane reactors can enable simultaneous H_2_S splitting and product separation, improving thermodynamic efficiency and simplifying downstream processing. Similarly, plasma‐assisted H_2_S splitting at atmospheric pressure offers rapid reaction kinetics and low‐temperature operation, but energy efficiency and electrode design require substantial refinement. Plasma‑based H_2_S decomposition combines relatively high energy demand with >90%–95% conversion, therefore making the production cost relatively lower, making it one of the strongest medium‑term candidates. Integration of H_2_S splitting into existing industrial infrastructure, such as desulfurization units in refineries or gas sweetening facilities, can enable on‐site H_2_ recovery while balancing H_2_S treatment costs.

The economic and environmental viability of low‐temperature electrochemical H_2_S conversion depends on how efficiently the system utilizes energy and integrates with industrial operations. Therefore, the targeted Faradaic efficiency could be set at >90%, where a current density of ∼100 mA cm^−2^ can be achieved at >1.5 V cell voltage. A promising frontier lies in indirect or hybrid systems. Tandem reactors where H_2_S is first chemically converted to polysulfides or organosulfur intermediates and then electrochemically valorized can offer such efficiency. Coupling electrochemical H_2_S oxidation with value‐added reduction reactions, such as CO_2_ or nitrate reduction, can ensure energy efficiency and economic co‐benefits. As global demand for clean H_2_ accelerates, electrochemical H_2_S splitting offers a compelling solution that bridges waste mitigation and energy production. Targeted research into catalyst design, system engineering, and scalable process configurations will be instrumental in unlocking its full potential.

In the realm of space exploration, H_2_S splitting is emerging as a critical consideration for future lunar missions. If lunar polar ice contains significant amounts of H_2_S as predicted by some models, then the ability to efficiently split this compound becomes paramount for extracting H_2_ locally, which will be a prime target for utilization of in situ resources of any space mission. Produced sulfur can be used in the construction of sulfur batteries. These are less developed for space applications and would significantly reduce the need for costly Earth‐launched resources, enabling more sustainable and ambitious long‐duration missions. Therefore, H_2_S splitting offers a specialized, energy‐efficient solution for H_2_ recovery from hazardous sources with the potential to unlock new resource streams, both industrially on Earth and extraterrestrially in space research. Cross‐disciplinary efforts combining catalysis, energy systems, and process intensification are expected to play a pivotal role in advancing this technology from lab to large‐scale deployment.

Table 5 highlights not only the operational distinctions among the major H_2_S to H_2_ conversion technologies but also the critical challenges that shape their practical feasibility. Thermo‑catalytic processes remain constrained by the need for very high operating temperatures, which drive up energy consumption and accelerate catalyst deactivation through sulfur poisoning and sintering [30, 32, 48]. These factors limit long‑term stability and significantly increase operational costs, especially in systems without integrated heat recovery. Photocatalytic approaches, despite their low‑energy requirements, face persistent issues with sulfur fouling and photo‐corrosion, both of which rapidly block active sites and diminish light absorption. This leads to short catalyst lifetimes and low durability under continuous operation, making large‑scale deployment difficult. Electrocatalytic systems, though highly efficient for H_2_ generation, are hindered by electrode passivation, polysulfide crossover, and membrane degradation issues that complicate cell maintenance and raise material costs. Finally, plasma‑assisted processes encounter challenges related to energy efficiency and scale‑up. Maintaining stable, uniform plasma at larger volumes is technically demanding, while high power inputs can offset the benefits of low‑temperature operation. Collectively, these challenges illustrate that while each technology offers compelling advantages, substantial materials innovations and engineering optimizations are still required before any of them can achieve widespread industrial implementation.

**TABLE 5:** H_2_S to H_2_ conversion technologies comparison and selection guidelines, highlighting the energy demands and performance characteristics that define each pathway.

Metric	ThermoCatalytic	Photocatalytic	Electrocatalytic	PlasmaAssisted
Energy Demand	High (endothermic, large heat input) [137]	Low–moderate (lightdriven)	Low–moderate (driven by applied potential; <0.5 V possible)	Moderate–high (depends on electron density and plasma power)
Sulfur Management	Solid S requires condensation or removal. Catalyst fouling is common	Sulfur fouling and photo‐corrosion major bottlenecks	Sulfur passivation of anode/cathode Polysulfide crossover	Elemental S condenses downstream Plasma‐catalyst surfaces can tolerate S
Catalyst Stability	Limited by sulfur poisoning and sintering	Often low due to sulfur deposition and photocorrosion	Good with sulfurtolerant catalysts (CoNi, Fe_2_NiSe_4_)	Good for sulfide semiconductors (ZnS, CdS, MoS_2_) Stable under plasma
Best Use Cases	High H_2_S concentration streams with available waste heat	Low temperature, renewable driven, small scale systems	High purity H_2_ needs, renewable electricity, refinery offgases	Dilute or variable H_2_S streams Remote or distributed sites

## 6 Current Market Status

1. Pilot Projects and Early‐Stage Development: Electrochemical H_2_S splitting technology is currently in the experimental and pilot project stages. While commercial water electrolysis has a stronger foothold in the market, H_2_S electrolysis is only recently being explored by some companies, particularly those within the oil and gas sectors.

2. Growing Interest in Oil‐Rich Regions: Regions with abundant H_2_S emissions, particularly those with large oil and gas operations, are increasingly exploring H_2_S conversion technologies as part of their decarbonization strategies. The Middle East, North Africa, and parts of North America are key hotspots due to their high volumes of sour gas and refinery off‐gases. Various oil and gas companies have initiated pilot programs to assess the viability of H_2_S‐to‐H_2_ conversion as a dual‐purpose solution, like mitigating sulfur emissions and generating clean H_2_. These initiatives align with broader national goals to diversify energy portfolios and reduce reliance on fossil fuels. In some cases, H_2_S splitting is being considered as a complementary technology to existing sulfur recovery units, potentially transforming waste streams into valuable H_2_ feedstock.

3. Technology Partnerships and Research: Collaborative efforts between industry players, academic institutions, and government agencies are accelerating the development of H_2_S conversion technologies. These partnerships are focused on overcoming key technical barriers such as reactor design, catalyst degradation, membrane fouling, and low conversion efficiency. Joint research programs are investigating advanced materials like transition metal sulfides, perovskites, and metal oxide catalysts to improve performance and reduce costs. Additionally, some partnerships are exploring modular reactor designs that can be scaled up or integrated into existing refinery infrastructure. Funding from energy innovation grants and sustainability‐focused venture capital is also helping to bridge the gap between lab‐scale research and commercial deployment. As these collaborations mature, they are expected to produce standardized systems that can be deployed across multiple industries, including petrochemicals, wastewater treatment, and geothermal energy.

4. With improvements in these areas, there is a significant advantage of H_2_S splitting in the production of elemental sulfur as a valuable byproduct. Sulfur has numerous industrial uses, including as an agricultural fertilizer and a key chemical feedstock. The sale of this sulfur byproduct provides an additional revenue stream, helping to offset production costs and enhance overall economic viability. This process effectively converts an environmental liability, H_2_S emissions, into a productive resource, promoting a circular economy. The global elemental sulfur market was valued at approximately $12.81 billion in 2024 and is projected to grow. Sulfur is used across various sectors, from the chemical industry, where it is converted into sulfuric acid, fertilizers, pesticides, detergents and rubber, to energy storage technologies like sodium‐sulfur and lithium‐sulfur batteries, which are gaining significant interest. This dual benefit of producing H_2_ and a valuable commodity makes H_2_S splitting an attractive solution for industries with abundant H_2_S, such as oil and gas production, with potential for larger‐scale adoption.

## 7 Challenges and Future Outlook

**FIGURE 7:**
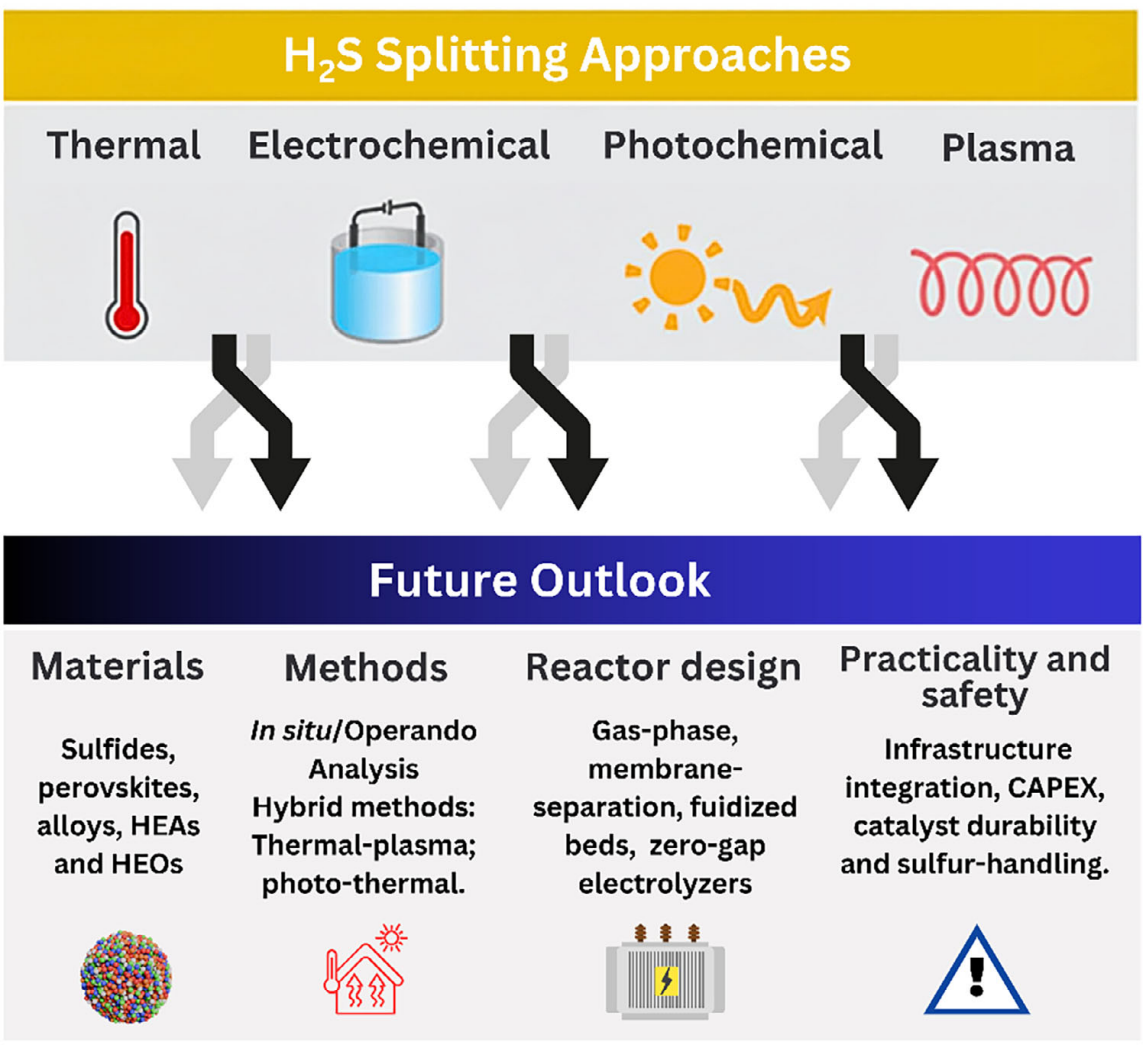
Summary of the future research directions on H_2_S to H_2_ production.

The main challenge in H_2_ production from H_2_S is related to the sulfur poisoning (or sulfur passivation) that arises because of the adsorbed S when deposited as solid sulfur or sulfides, strongly block active sites. Tackling this issue requires combining catalyst design along with smart control over process and reactor strategies that keep sulfur either weakly bound, dissolved, or continuously removed from the active interface. Across all approaches, the development of robust, sulfur‐tolerant catalysts is the central interest. Figure 7 summarizes the potential approaches to address these challenges and the direction of future research.

1. Challenges persist as H_2_S is highly corrosive, requiring robust materials and specialized catalysts that resist degradation, especially for the cathode, which significantly adds to capital costs. Advanced materials like transition metal sulfides and other corrosion‐resistant catalysts are being developed, but they come with a higher price tag, balancing the cost benefits of lower energy use. Utilizing advanced, corrosion‐resistant catalysts, such as CoFeS_2_, has demonstrated high efficiency and durability, crucial for reducing maintenance and replacement costs in a corrosive H_2_S environment. While the initial expense for these catalysts is higher, ongoing research shows potential for reduced costs as new materials become available. Additionally, the development of these catalysts is leading to electrolysis processes with up to 98% Faradaic efficiency, suggesting that durable and economical catalyst solutions are within reach for scaled production [138]. Durable materials like MoS_2_ and other metal dichalcogenides enable extended operational lifetimes by withstanding harsh sulfur environments. This durability minimizes replacement costs, a key consideration for long‐term H_2_ production at large scale. Research has shown promising long‐term stability (over 150 h in some studies) under continuous operation, which helps in lowering the cost per kilogram of H_2_ produced over time. Apart from single‐component catalysts, heterojunction structures often exhibit better physicochemical properties than individual components.

2. Thermocatalytic decomposition of H_2_S faces several technical and economic challenges that hinder its widespread adoption. The process requires high temperatures, usually above 800°C, to overcome the endothermic nature of the reaction, resulting in significant energy consumption and operational costs. Catalyst deactivation due to sulfur poisoning is another major issue, as sulfur tends to accumulate on active sites, reducing efficiency and necessitating frequent regeneration. Conversion rates are often limited by thermodynamic equilibrium, and even advanced reactor designs struggle to push yields beyond 50% in single‐pass systems. Material stability under corrosive and high‐temperature conditions also poses constraints on reactor construction and longevity. Looking forward, the development of sulfur‐tolerant catalysts such as transition metal sulfides, perovskites, and alloy‐based catalyst offers promising avenues to improve performance. Integrating thermocatalysis with membrane separation or sorption‐enhanced processes could help shift reaction equilibrium and boost H_2_ recovery. Hybrid systems that combine thermocatalysis with plasma may enable lower‐temperature operation while maintaining high conversion rates. Industrial integration into existing sulfur recovery units or refineries could further enhance the economic viability of this method, especially if coupled with heat recovery and process intensification strategies.

3. In liquid‐phase photocatalysis, optimization strategies are largely hindered by the competitive water‐splitting for H_2_. One of the major challenges is the deactivation of photocatalysts due to the accumulation of sulfur species on active sites, hindering light absorption and surface reactions. The fundamental redox reactions involved in H_2_S photocatalytic splitting result in the formation of elemental sulfur and polysulfides. Studies using in situ spectroscopy and surface analysis techniques have shown that catalyst deactivation is primarily due to sulfur fouling, which blocks active sites and reduces light penetration. To mitigate this issue, flowing gas‐phase reactors have been designed to continuously remove H_2_ and S, reducing fouling. Another approach is to use oxidative environments or electron acceptors (e.g., O_2_, Fe^3^^+^) that can convert S^0^ to soluble sulfate or sulfite, keeping the catalyst surface clean. pH of the reaction medium also controls the oxidative behavior of H_2_S. Formation of active species such as HS^−^ or S^2^^−^ depends on pH and significantly affects the reaction mechanism. Studies using in situ spectroscopy show the two‐step oxidation of sulfide ions to S^0^ and then to polysulfides. Short exciton/charge diffusion lengths and abundant surface traps lead to fast e^−^/h^+^ recombination, especially in high‐sulfur media. Polysulfide redox couples can act as electron/hole shuttles, further quenching useful activity. Attempts were made to design hydrophilic‐hydrophobic composite surfaces to prevent sulfur agglomeration on active sites, but they have still not achieved significant success.

4. Photothermal catalysis is a complementary emerging route. Photothermal catalysts convert light into localized heat (and sometimes plasmonic hot carriers), which can accelerate surface reaction kinetics and improve H_2_ production efficiency. Recent H_2_‐focused photothermal reviews highlight mechanisms such as localized surface plasmon resonance (LSPR), non‐radiative relaxation, and defect‐engineered hybrid catalysts concepts for H_2_O splitting, which can be translated to H_2_S splitting systems for improved activity and sulfur tolerance.

5. The electrolysis of H_2_S to produce H_2_ faces several challenges that impact its efficiency and feasibility. The corrosiveness of H_2_S can lead to significant degradation of electrode materials, necessitating the use of robust but potentially costly materials. Ensuring electrode efficiency is crucial, as the performance of catalysts affects reaction rates; however, side reactions can produce unwanted byproducts, complicating the process. Energy consumption is another concern, with high overpotentials sometimes required, which can make the process economically unviable. Safety is also paramount due to the toxicity of H_2_S, requiring stringent measures to protect workers and the environment. Addressing these challenges through ongoing research and development is essential for realizing the potential of this electrochemical method for sustainable H_2_ production. By combining low cell resistance with the high mass transfer performance of flow cells, membrane electrode assembly electrolyzers offer a persuasive route to high‐efficiency operation. However, fabrication of such a reactor is challenging and has yet to meet the industrial standards.

6. Electrochemical Reactor design is essential to achieve efficient electrochemical conversion. Zero‐gap electrolyzers and membrane electrode assemblies designed for H_2_S splitting. Use of flow‐through electrodes and segmented flow reactors would be useful to enhance mass transport and facilitate in situ sulfur removal. Implementation of periodic pulsed cleaning, or self‐cleaning electrode architectures using surface vibration or gas bubbling, would allow longer reactor life. Some other routes to achieve high conversion efficiency with reactor stability would be integration of in‐line sulfur removal units, such as heated traps or sulfur‐selective membranes, and use of hydrophilic/hydrophobic patterning to direct sulfur deposition away from active catalytic zones.

7. Although plasma technologies offer exceptional advantages, plasma‐based H_2_S conversion processes still require relatively high energy compared to traditional H_2_ production methods such as steam methane reforming (SMR). Achieving industrial‐level energy efficiencies needs further optimization of stable plasma generation, reactor design, and process integration. Most of the plasma processes for H_2_S conversion have been proven at the laboratory scale. Scaling up to industrial‐scale H_2_S volumes poses various technical challenges, such as reactor engineering, power supply, heat management, and product separation. In addition, environmental and regulatory requirements often demand>99% high sulfur recovery rates, which current catalytic systems struggle to consistently achieve at the industrial scale. Tackling these challenges will require innovations in catalyst design to resist sulfur poisoning, reactor engineering for effective heat and mass transfer, and approaches to reduce energy consumption and overall process costs.

8. In situ/operando spectroscopy is a powerful tool to understand the mechanistic details of the passivation mechanism and guide rational catalyst design. Integration with computational screening and machine learning is emerging to accelerate catalyst discovery. Reactor optimization, for example, membrane reactors, fluidized beds, with controlled heat and mass transfer, can minimize sulfur clogging and increase yield.

9. Recent efforts have begun exploring non‐noble metal catalysts, as well as interface‐rich heterostructures, that enable site‐specific H‐S bond activation while promoting the desorption of H_2_. In that aspect, high entropy alloys (HEAs) and oxides (HEAOs) are promising materials for advanced catalytic processes. Such catalysts with five or more principal metal elements in near‐equimolar ratios, offer a unique structural and compositional complexity that leads to exceptional thermal stability, corrosion resistance, and tunable electronic properties. HEAOs with their multicomponent oxide lattices provide enhanced oxygen mobility, defect engineering capability, and catalytic versatility. Moreover, smart conversion of HEAs or HEAOs to high entropy sulfide would be an interesting system for future study. Synergistic interactions among the multiple metal constituents, mixed valency, and high configurational entropy can lead to a high density of active sites and optimized adsorption energies for reactant molecules; therefore, they can significantly influence the reaction pathway for H_2_S splitting.

10. A deeper analysis of scalability and industrial integration reveals that each H_2_S conversion technology faces some constraints when interfacing with existing refinery and gas‑processing infrastructure, mostly the Claus process, which currently dominates global sulfur recovery. Thermo‑catalytic H_2_S decomposition is the closest to the operational conditions of the Claus process, but its integration requires redesigning reactor zones to accommodate catalytic beds, continuous sulfur removal, and heat‑integration loops. However, this faces various challenges, such as synchronized sulfur handling and corrosion‑resistant metallurgy compatible with 800°C–1200°C streams. Photocatalytic systems, despite their mild operating conditions, are limited by low throughput and sulfur‑fouling management. Electrocatalytic conversion, although promising for modular H_2_ production, requires robust mass‑transfer architectures for high‑flow sour gas streams, and membrane‑electrode assemblies struggle in the presence of polysulfides and particulates. Across all pathways, sulfur management remains a core integration bottleneck: solid S formed at different temperatures must be compatible with existing Claus condensers, sulfur pits, and degassing units. Overcoming these barriers will require hybrid configurations that couple H_2_‑recovery reactors with conventional Claus or SuperClaus units, advanced sulfur‑tolerant catalysts, corrosion‑resistant materials, and new control strategies capable of dynamically balancing H_2_‐recovery with regulatory requirements for >99% sulfur capture. While no single technology is immediately ready for full‑scale SRU replacement, these integration pathways represent realistic near‑term strategies that can bridge laboratory advances toward industrial deployment.

11. The practical deployment of H_2_S to H_2_ conversion technologies are ultimately governed by techno‑economic viability and operational safety, which remain key barriers to large‑scale adoption [139, 140]. From an economic perspective, the dominant cost drivers include energy consumption, reactor and materials capital expenditure, catalyst or electrode durability, and sulfur handling infrastructure. Thermo‑catalytic routes are burdened by high heat duties and materials requirements associated with sustained operation at elevated temperatures, although integration with waste‑heat recovery systems in refineries can partially offset these costs. Photocatalytic approaches benefit from low operating temperatures and potential use of solar energy, but their limited throughput and short catalyst lifetimes currently constrain economic competitiveness [141]. Electrocatalytic systems offer high H_2_ selectivity and low theoretical energy requirements. However, membrane costs, electrode passivation, and electrolyte management contribute significantly to operating expenses [139]. Plasma‑assisted technologies enable high conversion under non‑equilibrium conditions but require careful optimization of power efficiency and reactor design to reduce electrical energy costs at scale. Across all pathways, the co‑production and commercial value of elemental sulfur can provide an important revenue stream that partially offsets H_2_ production costs, particularly when integrated with existing sulfur recovery units [142].

12. Safety considerations are equally critical due to the extreme toxicity, corrosiveness, and flammability associated with H_2_S‑containing systems [143]. All conversion routes require stringent containment, continuous gas monitoring, and robust emergency shutdown protocols to mitigate risks associated with H_2_S exposure and H_2_ release [144, 145]. High‑temperature thermo‑catalytic systems pose additional risks related to material degradation, sulfidation corrosion, and thermal runaway, while electrochemical and aqueous chemical routes must address hazards linked to polysulfide crossover, acidic or alkaline electrolytes, and membrane failure [146]. Plasma‑assisted systems introduce electrical and electromagnetic safety challenges, including high‑voltage operation and localized hot spots [147]. Effective sulfur management is also essential to prevent solid sulfur accumulation, plugging, and pressure build‑up within reactors and downstream equipment [148]. Consequently, successful industrial implementation will depend on the co‑optimization of economics and safety through advanced materials, modular reactor designs, real‑time process control, and integration with established refinery safety and sulfur‑handling infrastructure.

## 8 Conclusion

The favorable thermodynamics and kinetics of H_2_S conversion make it an attractive route to H_2_ production and have urged the development of innovative materials and systems for high‐performance H_2_S splitting. To be economically and environmentally viable, these catalytic processes need to be carbon‐neutral and powered by sustainable, renewable energy. Although H_2_S conversion is a thermodynamically less energy‐demanding process, it still requires catalysts and reactors that are physically and chemically robust in harsh H_2_S‐rich environments. Key milestones include the development of long‐lived, sulfur‐tolerant catalysts; the elucidation of mechanistic pathways for H‐S activation and S evolution; and the demonstration of integrated systems at pilot scale. Future studies must include detailed life cycle assessment and techno‐economic analyses to evaluate the feasibility and environmental benefits of such integration strategies, and to assess the benchmark H_2_S splitting against conventional H_2_ routes. The state‐of‐the‐art materials, their current limitations, and possible pathways for rational catalyst design presented in this review will provide direction for future work upstreaming of H_2_S splitting for H_2_ research. With continued research and development, the sustainable and efficient use of H_2_S as a source of H_2_ and other value‐added chemicals could be realized at an industrial scale within the next decade.

## Conflicts of Interest

The authors declare no conflicts of interest.

## Data Availability

The authors have nothing to report.
